# Mass Spectrometry–Driven Discovery of Neuropeptides Mediating Nictation Behavior of Nematodes

**DOI:** 10.1016/j.mcpro.2022.100479

**Published:** 2022-12-05

**Authors:** Bram Cockx, Sven Van Bael, Rose Boelen, Elke Vandewyer, Heeseung Yang, Tuan Anh Le, Johnathan J. Dalzell, Isabel Beets, Christina Ludwig, Junho Lee, Liesbet Temmerman

**Affiliations:** 1 Animal Physiology & Neurobiology, Department of Biology, University of Leuven (KU Leuven), Leuven, Belgium; 2 Department of Biological Sciences, Seoul National University, Seoul, South Korea; 3 School of Biological Sciences, Queen’s University Belfast, Northern Ireland, United Kingdom; 4 Bavarian Center for Biomolecular Mass Spectrometry (BayBioMS), Technical University of Munich (TUM), Freising, Germany

**Keywords:** *Caenorhabditis elegans*, *Steinernema carpocapsae*, nictation, neuropeptidomics, parallel reaction monitoring, AGC, automatic gain control, CV, coefficient of variation, DDA, data-dependent acquisition, EPN, entomopathogenic nematode, FA, formic acid, IJ, infective juvenile, iRT, indexed retention time, LC-MS/MS, liquid chromatography coupled to tandem mass spectrometry, MS/MS, tandem mass spectrometry, m/z, mass-to-charge ratio, NGM, nematode growth medium, NLP, neuropeptide-like protein, PBS, phosphate-buffered saline, PRM, parallel reaction monitoring, RNAi, RNA interference, RNAseq, RNA sequencing, rpm, revolutions per minute, RT, retention time, UHPLC, ultra high performance liquid chromatography

## Abstract

Neuropeptides regulate animal physiology and behavior, making them widely studied targets of functional genetics research. While the field often relies on differential -omics approaches to build hypotheses, no such method exists for neuropeptidomics. It would nonetheless be valuable for studying behaviors suspected to be regulated by neuropeptides, especially when little information is otherwise available. This includes nictation, a phoretic strategy of *Caenorhabditis elegans* dauers that parallels host-finding strategies of infective juveniles of many pathogenic nematodes. We here developed a targeted peptidomics method for the model organism *C. elegans* and show that 161 quantified neuropeptides are more abundant in its dauer stage compared with L3 juveniles. Many of these have orthologs in the commercially relevant pathogenic nematode *Steinernema carpocapsae*, in whose infective juveniles, we identified 126 neuropeptides in total. Through further behavioral genetics experiments, we identify *flp-7* and *flp-11* as novel regulators of nictation. Our work advances knowledge on the genetics of nictation behavior and adds comparative neuropeptidomics as a tool to functional genetics workflows.

## Highlights

• Using mass spectrometry for the study of neuropeptides in nictation behavior.
• Novel discovery of neuropeptide genes brings the total to 97 in *Steinernema carpocapsae.*
• Application of parallel reaction monitoring for neuropeptide quantification.
• Neuropeptide genes *flp-7* and *flp-11* are identified as regulators of nictation.

## Graphical Abstract

**Figure:**
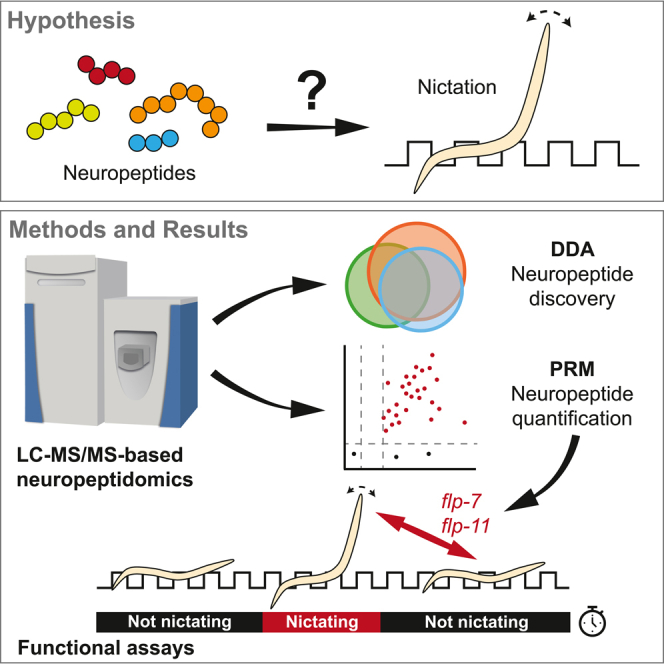

## In Brief

Neuropeptides are a type of signaling molecules that regulate certain behaviors. One such behavior is nictation, a strategy used by pathogenic nematodes for host finding. These pathogenic nematodes are often commercially significant, as they can be utilized as biopesticides in agriculture. Here, we have developed a method using mass spectrometry to identify and quantify neuropeptides responsible for nictation behavior. As a result, we were able to demonstrate that neuropeptide genes *flp-7* and *flp-11* are novel regulators of nictation.

Entomopathogenic nematodes (EPNs) are ubiquitous roundworms that infect and kill insect hosts with the help of symbiotic bacteria (1, 2, 3). Outside hosts, EPNs must survive without their food source and occur as infective juveniles (IJs), a nonfeeding and nondeveloping life stage actively looking for hosts. Host-finding strategies rely on two behaviors: dispersal by crawling, and ambushing, characterized by nictation. During nictation, a nematode lifts its body off the surface to stand on its tail, often also waving the body in three dimensions, allowing it to latch onto bigger animals that pass by (4). The actual combination of host-finding behaviors that is displayed depends on the preferred insect host of the nematode species or strain (5, 6, 7), suggesting there is genetic predisposition.

The ability of EPNs to infect a wide range of hosts and effectively kill them in a short time span has resulted in their application as biocontrol agents, protecting crops against insect pests. From the 1980s onward, EPNs have been commercialized as biopesticides, promoting further research into their biology to maximize their efficacy in the field (8). Although the virulence of commercialized EPNs is very high (6, 9, 10), host-finding behavior remains a critical step in the entire infection cycle. While efforts have been undertaken to unveil genetic regulation of host-finding behavior (5, 11, 12), there are but limited genetic tools available for EPNs and a model organism context would be useful. Nictation behavior, used by EPNs as part of ambushing behavior, is also observed in dauer juveniles of the free-living model nematode *Caenorhabditis elegans* (4), who use it as a hitchhiking strategy, enabling the rapid migration to a different (potentially nutrient-richer) environment (4). While the IJ stage of EPNs and the dauer juvenile stage of *C. elegans* are highly similar (13), the consequences of nictation are slightly different: in both cases, the behavior is food associated, but for *C. elegans*, it leads to phoresy, whereas for EPNs, nictation supports parasitism. Because nictation behavior itself and the life stage during which it is exhibited are similar, and because there is extensive genetic homology amongst nematodes (14, 15, 16, 17, 18, 19, 20), *C. elegans* may help to understand the genetics of nictation behavior in EPNs.

Amongst the possible genetically encoded regulators of nictation, neuropeptides are prime candidates. So far, three studies have causally linked specific neuropeptides (*flp-10*/*flp-17*, *flp-21*, and a subset of the insulin-like peptides) to nictation behavior (11, 21, 22). Several studies also found neuropeptide mRNA to be highly abundant in *C. elegans* dauers and in dauer-like stages of other nematodes, including EPNs (12, 21, 23). Furthermore, of the microRNAs that are more abundant in EPNs that also nictate more, several are predicted to regulate neuropeptide gene targets (12). Therefore, it can be expected that more neuropeptides may affect nictation, as part of the genetic mechanisms modulating phoretic and host-finding behaviors.

Here, we applied explorative and quantitative LC–MS/MS–based peptidomics and behavioral assays to prioritize and test neuropeptide candidates for their potential involvement in nictation. We probed the neuropeptide complement of the EPN *Steinernema carpocapsae* and developed a quantitative neuropeptidomics approach, enabling us to compare neuropeptide levels in *C. elegans* dauer juveniles and L3 stage worms. We show that IJs of *S. carpocapsae*, a popular EPN species, contain many endogenous neuropeptides, expanding its known neuropeptide complement to 97 genes. We find that most newly discovered and known neuropeptides in *S. carpocapsae* have orthologs in *C. elegans* and demonstrate a higher abundance of virtually all neuropeptides in dauer juveniles. Finally, we confirm the presence of neuropeptides that are known regulators of nictation (11, 21) and identify two additional neuropeptide precursor genes, *flp-7* and *flp-11*, as novel mediators of nictation.

## Experimental Procedures

### *C. elegans* Strains and Maintenance

The strains used in this work are described in supplemental Table S1. Worms were cultivated on nematode growth medium (NGM) at 20 °C (24) using 7.5 g/l peptone.

### *S. carpocapsae* Strains and Maintenance

The *S. carpocapsae* ALL strain was cultured in *Galleria mellonella* at 23 °C. Per infection, 5 to 10 wax moth larvae were placed in a petri dish containing filter paper at the bottom, which was slightly wetted with PBS (1.09 g/l Na_2_HPO_4_, 0.32 g/l NaH_2_PO_4_, 9 g/l NaCl, pH 7.4), making sure no puddles are formed. About 50 to 100 IJs (in PBS) per insect were dripped onto the filter paper, and the plate was sealed using parafilm (MLS). After 2 to 3 days, when all insects had died, cadavers were transferred to a White Trap (25). From 14 to 21 days after infection, IJs were collected and stored at 4 °C in petri dishes (in PBS). Fresh *G. mellonella* larvae were sourced from a local animal shop (https://www.terramania.nl/).

### *C. elegans* Dauer Generation

For behavioral assays, needing only a limited number of individuals, *C. elegans* dauers were generated *via* food deprivation (26) or pheromone treatment (27) of mixed cultures on NGM (7.5 g/l peptone) (see later). Peptidomics experiments require large numbers of individuals per sample (see further); for this, dauers were generated through overpopulation in synchronized liquid cultures (28) (see Sample Collection of IJs and Dauers for Peptidomics for protocol).

For dauer generation on NGM (7.5 g/l peptone), a preculture of *Escherichia coli* OP50 was grown in LB medium (37 °C) into the exponential phase (absorbance at 600 nm = 0.8). When at an absorbance at 600 nm of 0.8, a new culture was started by diluting the preculture 1:10 in LB medium at 37 °C, which was then grown to an absorbance of 1 at 600 nm, at which time, the bacteria were used for seeding NGM plates (100 μl per Ø 60 mm NGM plate). This standardized *E. coli* OP50 lawn was grown overnight (16 h) at 37 °C. Exactly 10 young adults were picked onto these plates, and these animals and their offspring were incubated at 20 °C for 8 days. This leads to starvation and overpopulation of the plate, thereby reliably inducing sufficient juveniles to enter the dauer stage. Dauers are easily identified by their radially constricted bodies and dark intestines and were picked off plates for use in behavioral assays using a capillary pipette (4) loaded with S basal (5.85 g/l NaCl, 6 g/l KH_2_PO_4_, and 1 g/l K_2_HPO_4_).

A second approach was based on combining pheromone exposure with increased temperature (4). Ten young adults were transferred to synthetic pheromone plates consisting of agar (10 g/l), agarose (7 g/l), NaCl (2 g/l), KH_2_PO_4_ (3 g/l), K_2_HPO_4_ (0.529 g/l), cholesterol (8 mg/l), supplemented with ascaroside C7 (ascaroside 3, daumone 1), ascaroside C6 (ascaroside 1, daumone 2), and ascaroside C9 (ascaroside 2, daumone 3) at 2 mg/l each (27, 29, 30). These were seeded with *E. coli* OP50 and grown overnight at 25 °C. Worms were incubated for 4 days at 25 °C on these seeded synthetic pheromone plates, after which dauers (identified by their dark intestines and radially constricted bodies, ∼80% of the population (4)) can be picked off for use in behavioral assays.

### Nictation Assays

Nictation was evaluated by observing the locomotion behavior of a single *C. elegans* dauer on a molded textured agar surface, also referred to as micro-dirt chip, essentially as described before (4). A 40 g/l agar solution was poured onto a polydimethylsiloxane mold containing the desired texture (a 610 × 610 matrix of 25 μm radius pillars of 25 μm high, spaced 25 μm apart, based on the design of Lee *et al.* (4)). After the agar solidifies, the micro-dirt chip was peeled off and dried for 90 min at 37 °C. About 50 dauers were collected with S basal and a capillary pipette and mounted on the chip, which ensures that at least 15 dauers can be accurately measured, as several dauers/IJs leave the micro-dirt surface. Worms were left undisturbed for 30 min so that transferred traces of S basal buffer may evaporate. Individual dauers/IJs were observed for 90 s, measuring the time they were nictating *versus* crawling and only including consistently moving dauers. Three nictation parameters were calculated as described by Lee *et al.* (31): nictation ratio (T_nict_/T_total_), initiation index (N_nict_/[T_total_ − T_nict_]), and average duration (T_nict_/N_nict_) of nictation, where T_nict_ is the total observed time a nematode was nictating, T_total_ the total observed time, and N_nict_ the number of nictation events. An in-house R script using publicly available libraries (32, 33, 34, 35, 36, 37) was used to calculate these parameters and analyze the data. Data were fitted to a linear model, and *p* values were calculated using a Dunnett’s test for multiple comparisons of means.

### *C. elegans* Synchronization

*C. elegans* populations were synchronized using hypochlorite treatment. When grown on plates, mixed culture populations were washed off using S basal into 15 ml tubes. When grown as liquid culture, the population was pipetted into 15 ml tubes. Then, these tubes were centrifuged at 350*g*, and the supernatant was aspirated until 3.5 ml were left. About 1.5 ml bleach solution (1 part 5 M NaOH, two parts 4% household bleach) were added, and tubes were shaken vigorously for 4 min. About 10 ml S basal were added, and samples were centrifuged at 350*g*. Pellets were then washed three times with S basal, centrifuging at 350*g* between each wash step. In case of bleaching for experiments to track locomotion of L3 juveniles, only small amounts of eggs were needed, and tubes were topped up with S basal to 10 ml and collected eggs were allowed to hatch overnight, in a rotator at 20 °C. In the case of sample preparation for quantitative peptidomics, eggs were diluted to specific densities (in S basal, specified later) and allowed to hatch in a baffled flask in a shaking incubator at 20 °C.

### Tracking L3 Locomotion

Locomotion of L3 juveniles was analyzed using an automated camera setup. First, worms were synchronized (as described previously) using hypochlorite treatment, and embryos were left to hatch overnight at 20 °C in S basal, in the absence of food. Locomotion of L3 juveniles was tracked 29 h after introducing the synchronized L1 juveniles to OP50-seeded NGM plates.

L3 juveniles were washed off plates using S basal and allowed to settle for 15 min at 20 °C. Approximately 50 L3 juveniles were dropped onto an unseeded NGM plate in as little liquid as possible. Excessive liquid was removed using lint-free Kimwipes, and the plates were left to dry for 5 min to allow any possible leftover liquid to evaporate. Then, worms were recorded for 30 min using either one of two in-house setups: (1) as described by Watteyne *et al.* (38) and using the same MATLAB (MathWorks) script to track worms or (2) as described here, using a grayscale machine vision camera (Imaging Source DMK27AUP031), 50 mm lens (Kowa LM50JC10M), acrylic glass stage, and bright field illumination source. The illumination source consisted of a flat matrix of white light-emitting diodes with a diffuser. The incidence angle of illuminating light was constrained with a 22 mm inner diameter lens tube (RICOH FP-RGST; Stemmer-Imaging) and two layers of privacy screen filter taped to the underside of the stage. The field of view was approximately 5.0 × 3.7 mm with a resolution of 2560 × 1920 pixels. An in-house script was used to track worms. Using either script, worm behaviors (crawling, pausing, and omega turns) were determined for all worm tracks (minimal length of 30 s), and the average crawling speed was calculated per track. Speeds in pixels/seconds were scaled to micrometer/seconds so that data from both imaging setups could be compared. Data were averaged per experiment, fitted to a linear model, and *p* values were calculated using a Dunnett’s test for multiple comparisons of means.

### Collection of Mixed-Stage wildtype Samples for Peptidomics Method Development

Three mixed-stage liquid cultures of *C. elegans* wildtype worms were prepared each by rinsing worms off a 90 mm NGM plate (seeded with OP50) and adding the animals to a sterile baffled Fernbach flask containing 1 l of S medium. S medium is generated by supplementing 1 l of S basal with 10 ml of 1 M potassium citrate (20 g/l citric acid monohydrate, 293.5 g/l tripotassium citrate monohydrate, adjusted to pH 6.0), 10 ml trace metal solution (1.86 g/l disodium EDTA, 0.69 g/l FeSO_4_.7H_2_O, 0.2 g/l MnCl_2_.4H_2_O, 0.29 g/l ZnSO_4_.7H_2_O, and 0.025 g/l CuSO_4_.5H_2_0), 3 ml CaCl_2_ (1 M), 3 ml MgSO_4_ (1 M), 1 ml cholesterol (5 mg/ml in ethanol), 5 ml penicillin/streptomycin/neomycin (Gibco), and 5 ml nystatin (Sigma–Aldrich). These cultures were grown at 20 °C under continuous shaking at 121 rpm (Eppendorf New Brunswick Innova 43R) and kept fully fed by daily supplementation with frozen *E. coli* K12 pellets as needed, maintaining an absorbance at 600 nm of 1.8. As soon as the worm density reached ∼1 worm per μl (after approximately 7 days), the three cultures were pooled in a large glass cylinder (12.5 cm diameter) with a conical bottom (tapering over a distance of 14 cm), in which worms were allowed to settle while kept on ice. The total worm pellet was collected in 15 ml tubes and subsequently washed three times with S basal, removing the supernatant each time after centrifugation at 350*g* for 3 min. The washed worm pellet (approximately 10 ml) was aliquoted per 1 ml in microcentrifuge tubes, flash-frozen in liquid nitrogen, and stored at −80 °C, awaiting peptide extraction.

### Sample Collection of IJs and Dauers for Peptidomics

For the neuropeptide discovery samples in *S. carpocapsae*, IJs were collected as described previously. Then, IJs were washed three times in PBS buffer, centrifuging at 350*g* between each wash step, and pelleted at 350*g*. These were flash frozen as aliquots of 0.3 ml or 0.5 ml and stored at −80 °C until peptide extraction. After homogenization (see Peptide Extractions section for details), samples were made by combining aliquots of 0.3 ml pellet into a 0.6 ml sample, a 0.9 ml pellet sample, a 1.8 ml pellet sample, and a 3.9 ml pellet sample. The 0.5 ml pellets were combined into three samples, each containing 3 ml of pellet.

Dauers for neuropeptide extraction samples for quantitative comparisons were grown as synchronous populations in liquid (see summary, supplemental Fig. S1). For each replicate, a starter culture (P0) of 15 NGM plates (Ø 90 mm) with each 10 young adult *C. elegans* was prepared. After 4 days (96 h, F1) of incubation at 20 °C, eggs were collected by hypochlorite treatment as described previously and hatched overnight in S basal at 10 eggs/μl (F2). The next day, 150,000 synchronized L1 juveniles per sample were further cultured in a liquid preculture (as described previously, 150 ml in a 2 l baffled flask) at one worm per microliter for another 3 days (until adulthood, 72 h), which was bleached and synchronized again by hatching overnight in S basal at 10 eggs/microliter in a baffled flask (F3). To obtain the sample cultures, this population was split over two conditions: a fully fed population that was sampled at the L3 stage (10^6^ worms at one worm per microliter, grown for 34 h at 20 °C, shaking at 121 rpm in 2× 500 ml in a 2 l baffled flask) and an overpopulated culture that was starved (*i.e.*, not refed after the initial provided food) and sampled as dauers (10^6^ worms at 20 worms per microliter, grown for 72 h in 50 ml in a 0.5 l baffled flask). L3 juveniles were collected by letting the worms settle in a large glass cylinder (12.5 cm diameter) with a conical bottom (tapering over a distance of 14 cm) on ice. Dauers did not need settling, since they were sufficiently concentrated. L3 and dauer samples alike were washed three times with S basal, centrifuging at 350*g* between each wash step and then concentrated (by centrifuging at 350*g*) and aliquoted into three 500 μl pellets per culture, each pipetted into a Precellys 2 ml tissue homogenizer tube (Bertin Instruments). These were flash frozen in liquid nitrogen and stored at −80 °C until peptide extraction. All liquid *C. elegans* cultures were supplied with *E. coli* HB101 as a food source to a concentration of 1.98 absorbance at 600 nm. Bacterial concentrations in liquid cultures were verified twice daily and adjusted to this absorbance when necessary with *E. coli* HB101 from a frozen (−80 °C) stock, previously grown in Super Broth (0.017 M KH_2_PO_4_, 0.072 M K_2_HPO_4_, 12 g/l bactotryptone, 24 g/l yeast extract, and 8 ml/l glycerol) and collected after 16 h of incubation at 37 °C (except for the dauer samples, these were only fed once at the beginning and then left to starve).

### Peptide Extractions

To extract peptides, we used an in-house developed method based on acidified methanol as described previously (39, 40, 41, 42, 43, 44), with small changes dependent on the sample type, detailed later.

For discovery samples (*S. carpocapsae* IJs), we pooled multiple frozen pellets to 0.6, 0.9, 1.8, 3× 2.9, and 3.9 ml to cover an as-wide-as-possible concentration range of detectable neuropeptides. Frozen pellets were pulverized in liquid nitrogen using mortar and pestle. Then, acidified methanol (precooled on dry ice) was added to a final concentration of 90:9:1 methanol/worm pellet/acetic acid. Samples were sonicated four times for 15 s with intermittent 30 s cooling on dry ice (Branson Ultrasonic SLPe; 80% amplitude). After centrifugation (2100*g*, 12 min), samples were transferred and distributed over 2 ml tubes, and methanol was evaporated using a Thermo Savant SpeedVac vacuum concentrator. After collection of concentrated sample content, samples were diluted to 4 ml using 0.1% formic acid (FA) in water. Samples were delipidated three times using 2 ml *n*-hexane. The peptide fraction was then purified using an Amicon Ultra-4 10 kDa filter (Merck Millipore) as described by the manufacturer. Finally, samples were concentrated using Pierce C18 spin columns according to the manufacturer’s instructions and using FA for acidification. Samples were dried (Thermo Savant SpeedVac), redissolved in 5% acetonitrile with 0.1% FA, and stored for 1 to 3 weeks at 4 °C prior to analysis.

For quantitative samples (synchronous *C. elegans* L3 and dauer juveniles), 500 μl of 1.4 mm ceramic beads (zirconium oxide; Bertin Instruments) were added to each pellet together with 1 ml of 90/9/1 methanol/Milli-Q water/acetic acid and then homogenized for 10 cycles of each 15 s at 6800 rpm with 45 s rest, in a Precellys homogenizer with Cryolys cooling unit (kept at −15 to −25 °C; Bertin Instruments). Of the three frozen aliquots per liquid culture, two were homogenized and then combined to continue with 1 ml biological material per sample. Each sample was topped up to 10 ml with the 90/9/1 solution. Further extraction was performed as of the sonication step as described previously. For retention time (RT) calibration, indexed RT standard peptides (Biognosys AG) (45) were added as specified by the manufacturer’s protocol. Samples were dried (Thermo Savant SpeedVac) and stored at −80 °C until use (1–3 weeks), when they were dissolved in 45 μl of 2% acetonitrile with 0.1% FA prior to analysis.

The mixed-stage samples that were used for developing the peptidomics method were done as described for the quantitative method, except using a glass homogenizer instead of the Precellys homogenizer. Aliquots of these mixed-stage samples were used to validate the developed parallel reaction monitoring (PRM) method by spiking in the *C. elegans* synthetic neuropeptides (identical to those used for spectral library generation). This allowed us to estimate the effects of a biological matrix on peptide RTs and to validate the success of the PRM method on a sample where all neuropeptides are present. No synthetic peptides were spiked into the L3 *versus* dauer juvenile samples, except for the Biognosys iRT standards, which are not biologically present in *C. elegans.*

### LC–MS/MS

For peptide discovery, samples were analyzed on a Dionex UltiMate 3000 UHPLC coupled online to a Q Exactive mass spectrometer (Thermo Scientific). The UHPLC was equipped with a guard precolumn (Acclaim PepMap 100, C18, 75 μm × 20 mm, 3 μm, 100 Å; Thermo Scientific) followed by an analytical column integrated in the nano-electrospray ion source (EASY-Spray PepMap RSLC C18, 50 μm × 150 mm, 2 μm, 100 Å; Thermo Scientific). The solvent flow was set to 300 nl/min with a 75 min linear gradient from 4% to 36% acetonitrile, always containing 0.1% FA. Samples were injected in the mass spectrometer using its nano-electrospray ion source, and data were acquired in a data-dependent manner (Top10 method, dynamic exclusion: 10 s), always selecting the most abundant precursor ions with charges [+2, +5] from a full mass spectrometry (MS) survey scan for higher-energy collisional dissociation fragmentation. Full MS1 scans were acquired at a resolution of 70,000 at an *m/z* 200, with automatic gain control (AGC) set at 3e6, a maximum injection time of 256 ms, and scan range of 400 to 1600 *m/z*. The resolution for the MS/MS scans was set at 17,500 at *m/z* 200, with an AGC of 1e6, maximum injection time of 64 ms, and normalized collision energy of 25.

For the label-free quantification of *C. elegans* neuropeptides, targeted PRM measurements were performed on a Dionex UltiMate 3000 UHPLC coupled online to a Q Exactive HF-X mass spectrometer (Thermo Scientific). Two separate injections were performed, each time loading 5 μl of sample on a trap column (ReproSil-pur C18-AQ, 5 μm, Dr Maisch, 20 mm × 75 μm, self-packed) at a flow rate of 5 μl/min in 0.1% FA. After 10 min of loading, samples were passed onto the analytical column (ReproSil Gold C18-AQ, 3 μm, Dr Maisch, 450 mm × 75 μm, self-packed), from which they were eluted at a flow rate of 300 nl/min, using a 50 min linear gradient from 4 to 32% solvent B (0.1% FA and 5% dimethyl sulfoxide in acetonitrile) in A (0.1% FA in 5% dimethyl sulfoxide). Analytes eluting from the HPLC undergo electrospray ionization right before introduction into the MS instrument.

Full scan MS1 spectra were recorded in the 360 to 1300 *m/z* range with a resolution of 60,000 at 200 *m/z*, using an AGC target of 3e6 and maximum injection time of 100 ms. A normalized collision energy of 26 was used for higher-energy collisional dissociation, and targeted MS2 spectra were acquired with a resolution of 15,000 at 200 *m/z*, an AGC target value of 1e6, a maximum injection time of 22 ms, and an isolation window of 0.9 *m/z*. Depending on the run, the number of targeted precursors was adjusted to not exceed 50 or 70 to 80 simultaneous targets at a given RT. The RT windows used for the scheduled PRM experiments ranged from 5 min for the *C. elegans* technical replicates, to 10 min for the *C. elegans* L3 *versus* dauer juvenile comparison. The higher biological variability between replicates motivates this widening of RT windows.

### MS Data Analysis

For the peptide discovery samples, raw data files were analyzed using PEAKS Studio X+ (version 10.5; Bioinformatics Solutions, Inc). For *de novo* searches, parent mass error was set at 10 ppm, with a fragment mass error of 0.04 Da. Enzyme was set to “none,” and the following variable post-translational modifications were taken into account: oxidation of methionine (+15.99 Da), pyroglutamation of N-terminal glutamic acid (−18.01 Da) or glutamine (−17.03 Da), C-terminal amidation (−0.98 Da), and half of a disulfide bridge on cysteine (−1.01 Da). For PEAKS DB searches, the same error tolerances and post-translational modifications were applied. Here, the enzyme parameter was again set to “none” and digest mode to “unspecific.” Since two versions of the *S. carpocapsae* proteome exist (46, 47), a union of both databases was used as a search space for PEAKS DB. A false discovery rate of <1% was applied to MS/MS peptide identifications, and .csv files containing the detected peptide sequences and database IDs were exported for further analysis using R (R Core Team, 32, 34, 36, 37, 48). Potential neuropeptide precursors were identified based on (1) peptides that have flanking dibasic peptide cleavage sites (arginine and/or lysine, as predicted by NeuroPred (49)) and (2) the presence of a signal sequence at the N terminus of the precursor protein, as predicted with SignalP 5.0 (50). Full details (precursor charge, measured *m/z*, and scores) of all neuropeptides detected in *S. carpocapsae* are outlined in supplemental Table S2. All raw data are available *via* the PRIDE data repository with dataset identifier PXD034629.

For quantitative analysis, first a *C. elegans* neuropeptide spectral library had to be created. An in-house collection of 427 synthetic neuropeptides, making up the known and predicted *C. elegans* neuropeptidome (51), was subjected to data-dependent acquisition (DDA) on a Dionex UltiMate 3000 coupled online to a Q Exactive HF-X mass spectrometer and analyzed with PEAKS Studio X+ using the same settings as described earlier. A spectral library was built from these data using Skyline-daily (64 bit, version 21.1.1.223 (52)). The quality of the spectral library was manually evaluated, taking into account data points across the peak, peak shape, and dotp value.

All PRM data were analyzed using Skyline-daily (64 bit, version 21.1.1.223). For all target neuropeptides, the most intense precursor charge state and the six most intense fragment ions were selected automatically by Skyline from the neuropeptide spectral library. Raw PRM data were imported into Skyline and reviewed: if necessary, integration boundaries were manually adjusted, and strongly interfering transitions were removed from the dataset, keeping at least five transitions per peptide. Peptide ion peaks were only retained for differential analysis when detected in all replicates of both the L3 and dauer conditions, or when detection was limited to all replicates of only a single condition (reflecting on/off-type differences between conditions). Since the absence of a suitable peak to integrate results in missing values (NA, not available), integration boundaries for signals below the detection limit were set around similar RTs as those in samples in which they were successfully detected, effectively integrating the background signal. In some cases, this resulted in a zero value (0) because of a flatline signal. In these cases, any remaining zero values for the area under the curve of the fragment ions were replaced by the minimum value of the entire dataset (53). Full details (precursor charge, *m/z*, RTs, and scoring) of all the neuropeptides quantified in *C. elegans* L3 and dauer juveniles using PRM are available *via* the Panorama Public data repository *via* this direct link: https://panoramaweb.org/targeted_neuropeptidomics-L3_vs_dauer.url.

All data were exported from Skyline and further analyzed with R (32). In cases where multiple ions were observed because of different charge state or the presence of an oxidized methionine, the total fragment area of all the ions was summed. Subsequently, fragment peak areas of each run were normalized using the median abundance of all MS1 features. After log_10_ transformation, the peptide precursor abundancies of the individual peptides were compared, and significance was determined using a Student’s *t* test.

The L3/dauer PRM data were used as the data source for power analysis. From the log_10_ transformed data, the mean and standard deviation were calculated for each neuropeptide in each condition. With these statistics, data were simulated in R for a range of different effect sizes (fold changes of 1.1, 1.2, 1.5, 2, and 4) and sample sizes (4, 6, 8, 10, 15, and 20 replicates). A total of 500 simulations were performed for each of the 30 possibilities (five effect size parameters × six sample size parameters) per individual neuropeptide (n = 164), and the percentage of simulations that produced a significant result was calculated.

### Peptide Homology Search Using BLASTp and Neuropeptide Alignments

Command line BLASTp (54) was used to batch process selected sequences, confirm orthologs, and identify new orthologs of known neuropeptides. *S. carpocapsae* proteomes PRJNA202318.WBPS12 (28,313 genes, 31,944 transcripts (46)) and PRJNA202318.WBPS17 (30,931 genes, 36,703 transcripts (47)) were downloaded from WormBase Parasite (https://parasite.wormbase.org/). The BLOSUM62 scoring matrix was used, with a gap opening penalty of 11 and extension penalty of 1. An E-value threshold of 1e-2 was set as cutoff, and sequence identity was always manually verified.

Neuropeptides were aligned using the Clustal Omega (55) command line tool. Alignments were visualized using Jalview (56) and further formatted in Inkscape (Inkscape Team, 57).

### Experimental Design and Statistical Rationale

For the nictation assays, 5 or 10 biological replicates were used, based on several prominent nictation papers (4, 21, 22). As a rule of thumb, we performed five replicates, but since some mutant conditions displayed high variation, the number of replicates for these conditions was increased to 10 (with at least 15 nictation measurements per replicate; for exact n-numbers, see supplemental Tables S8 and S9). Assays were performed in batches for different conditions (each time with wildtype worms as the reference), and statistical analysis was performed on the individual worms, per batch. For ease of visualization, all batches are shown together in Results section.

Tracking and L3 locomotion assays were performed with 4 to 12 biological replicates. As with the nictation assays, there was high variation for some conditions, for which more replicates were used (for exact n-numbers, see supplemental Table S10).

The setup of the initial PRM experiments was designed with four replicates for testing variation during sample preparation. This number was motivated by standard proteomics workflows, where three to five replicates are common practice (58). With a median CV of 21.63 ± 1.63 (SEM), technical variation because of sample preparation was found to be within limits for (neuro)peptides. For biological variation, power analysis on the L3 *versus* dauer juvenile quantification (supplemental Fig. S4) confirmed that four biological replicates provided ample statistical power for the observed effect sizes (further elaborated in Discussion section and supplemental Fig. S4). Hence, for the quantification of changes in neuropeptide abundance in L3 and dauer juvenile samples of *C. elegans*, four biological replicates each were used.

Full details on the statistical data analysis for all experiments are described per experiment type in the previous paragraphs.

## Results

### *S. carpocapsae* IJs Contain a Rich Set of Neuropeptides

**Fig. 1:**
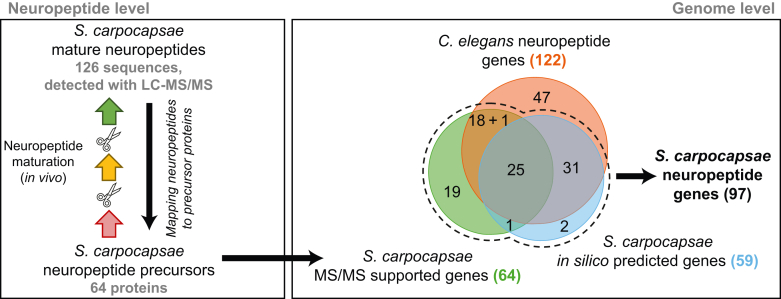
**Discovery-driven LC–MS/MS shows that neuropeptides are abundantly present in *Steinernema carpocapsae*–infective juveniles.***In vivo*, neuropeptides mature through a series of processing steps (*left panel*, *colored arrows*), including cleavage out of larger precursor proteins that typically contain several neuropeptide sequences. Using LC–MS/MS in data-dependent acquisition (DDA) mode, 126 of these mature neuropeptide sequences were identified and then mapped back to 64 neuropeptide precursors in *S. carpocapsae* (*right panel*, *green set*; details in supplemental Table S3). Of these, 25 overlap with our *in silico* predicted neuropeptide precursor set (*right panel*, *blue set*; details in supplemental Table S3), and many have clear sequence homologs in the annotated *Caenorhabditis elegans* neuropeptidome (*right panel*, *red set*; details in supplemental Tables S3 and S4). Of the genes for which we provide MS/MS support (*right panel*, *green set*), 19 had no known homologs in other nematodes and are here for the first time annotated as neuropeptide genes in *S. carpocapsae* (*nlp-100* to *nlp-118*). Another 19 do have clear *C. elegans* homologs, denoted as “18 + 1” in the diagram since one was not yet annotated as a neuropeptide-like protein in *C. elegans* prior to this work (*nlp-99*). Only one was found to have a homolog in other nematodes, but not *C. elegans*, and has been named *nlp-97*. In addition to the 59 *in silico* predicted neuropeptide genes of *S. carpocapsae*, we here unveil 38 genes as *S. carpocapsae* neuropeptide-encoding genes for the first time, expanding the neuropeptide complement to a total of 97 genes in this species.

To identify neuropeptide candidates that may regulate nictation, we set out to identify which neuropeptides are present in IJs, as nictation is only displayed in this life stage. LC–MS/MS was used to analyze whole mount extracts of *S. carpocapsae* IJs. We detected 126 peptides (0.1% false discovery rate) mapping to 64 precursor genes; 39 of these proteins are here for the first time identified as neuropeptide precursors in *S. carpocapsae* (5, 17) (Fig. 1 and supplemental Tables S3 and S4).

Among those 39 novel neuropeptide precursors, 18 are easily recognized because of a clear *C. elegans* neuropeptide gene homolog (*green–red* overlap in Figs. 1, S2 and supplemental Tables S3 and S4). One additional gene (“+1” in the *green–red* overlap of Fig. 1) seems homologous to T05A8.3 (supplemental Table S7), a *C. elegans* gene that was previously not annotated as a neuropeptide but which has a number of characteristics typical of a neuropeptide precursor gene. These include an open reading frame that starts with a signal peptide and a mature peptide that is flanked by two dibasic cleavage sites or by the signal peptide and a dibasic cleavage site. Based on these features, we propose to name this gene *nlp-99.* For one neuropeptide, we were able to find homologous sequences annotated as neuropeptides in other nematodes (*blue–green* overlap in Fig. 1 and supplemental Table S3, *nlp-97*) (17). For the 19 others (*green only* in Fig. 1), there is no nematode homolog already annotated as a neuropeptide gene, but manual inspection of the genes reveals they carry all the neuropeptide characteristics as described previously (Figs. 1 and S2). These 19 genes had not yet been recognized as neuropeptide genes, and we propose to annotate these new neuropeptide-like protein genes as *nlp-100* through *nlp-118* (supplemental Tables S3–S5). Of the 64 detected precursors, 25 matched to genes already annotated as neuropeptide-encoding genes in *S. carpocapsae* (intersection of all three sets in Fig. 1) (12). The known nematode neuropeptide complement (*C. elegans* (51) and other nematodes (17)) was also used as a query for BLAST to identify more non-MS detected neuropeptides in the *S. carpocapsae* proteome. Seventy-five of all *C. elegans* neuropeptide precursors seem to have a homolog in *S. carpocapsae*, and we found two additional non-*C. elegans* neuropeptide-like proteins (*nlp-93* and *nlp-*95), which were predicted by McKay *et al.* (Fig. 1 and supplemental Table S3) (17).

Combining the MS and *in silico* results, we here expand the known *S. carpocapsae* neuropeptide complement from 54 genes (12) to 97 genes. Based on these BLASTp results, 19 of the newly annotated genes do not seem to have orthologs in *C. elegans*, whereas *nlp-99* might be a novel *C. elegans* neuropeptide gene as well (+1 in the *green–red* overlap in Fig. 1 and supplemental Tables S3 and S4).

### PRM Permits Robust Quantification of the Majority of the *C. elegans* Neuropeptidome, Which Proves Generally Highly Abundant in Dauers

Since nictation is an IJ/dauer-specific behavior (4), we hypothesized that neuropeptides involved in the mediation of nictation behavior would likely be more abundant during this stage. Hence, relative quantification of the neuropeptidomes of *S. carpocapsae* IJs and J3 animals would provide interesting targets for further analysis. Unfortunately, the entire development of *S. carpocapsae* takes place inside the infected host, complicating the isolation of J3s for sampling. By contrast, many more tools are available for the model organism *C. elegans*, whose development can be timed precisely and whose dauers are very similar to IJs. This is true at the level of their morphology (both are small and radially constricted alternative third juvenile stages (13)), behavior (including the use of nictation as a food-searching strategy (59, 60)), and genome, as many neuropeptide genes have very similar sequences (Figs. 1, S2, supplemental Tables S3 and S4; (61)). For these reasons, we turned to *C. elegans* to examine changes in the L3 *versus* dauer juveniles neuropeptidomes, aiming to use these data to prioritize candidates and study their relation to nictation further. Approaches for the targeted quantification of bioactive peptides have currently been relatively small scaled, typically targeting only ∼10 peptides in a single experiment (62, 63). Since no comprehensive method yet exists for the simultaneous quantification of several hundred neuropeptide abundancies in biological samples, we first developed such a method for *C. elegans*, using a PRM workflow for LC–MS/MS.

First, synthetic versions of all 427 known and predicted neuropeptides in *C. elegans* (51) were subjected to a DDA LC–MS/MS workflow, yielding an extensive spectral library of 510 peptide ions, representing a total of 300 neuropeptides that are available for targeted LC–MS/MS. Consequently, approximately ∼70% of the known and predicted *C. elegans* neuropeptides are targeted with our method, providing a broad coverage of the neuropeptidome.

**Fig. 2:**
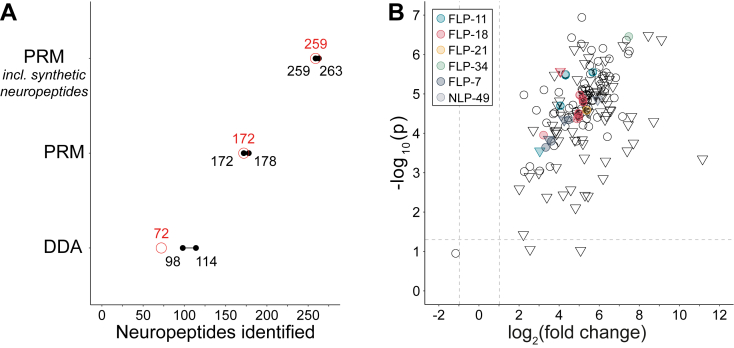
**Development and application of a parallel reaction monitoring LC–MS/MS method for neuropeptide quantification, indicating that neuropeptides are highly abundant in the dauer stage.***A*, comparison of DDA and PRM methods on four technical repeats of *Caenorhabditis elegans* mixed-stage neuropeptide extracts. The range of neuropeptides detected is indicated in *black*, and sample overlap is indicated in *red*. PRM increased both the absolute number of detected neuropeptides (+139%) and sample overlap (63% → 97%). Testing the method on biological samples spiked with synthetic versions of all neuropeptides indicates that it is 88% effective in detecting the targeted neuropeptides in a biological sample matrix. *B*, volcano plot showing that the vast majority of mature neuropeptides is much more abundant in *C. elegans* dauers *versus* L3 juveniles. Triangles (∇) depict neuropeptides whose detection was restricted to dauer juveniles. *Circles* (○) were detected in both sample types. Neuropeptides indicated with a color were tested in subsequent assays. DDA, data-dependent acquisition; PRM, parallel reaction monitoring.

Using RT scheduling, we were able to reduce the number of sample injections required to quantify all target ions to 2, compared with 13 injections when using an unscheduled LC–MS/MS workflow, making it more manageable and requiring far less sample. We next validated the synthetic peptide–based assays on four technical replicate extracts of a mixed-stage *C. elegans* culture, assuming that diversity of the population making up the sample would benefit diversity of the observable neuropeptidome in these test samples. With the two-injection scheduled PRM method, we detected between 172 and 178 quantifiable neuropeptides per sample (Fig. 2*A* and supplemental Table S5), with 172 neuropeptides being detected in all four (∼97% sample overlap). As expected, this is in stark contrast to a standard DDA method (51, 64), with which between 98 and 114 neuropeptides could be detected (but not quantified) in these same samples, with only 72 being detected in all four (∼63% sample overlap, Fig. 2*A* and supplemental Table S6). When comparing the number of robustly identified neuropeptides in all samples for DDA (72 neuropeptides) and PRM (172 neuropepides), we report an increase of 139% in identification potential.

To verify that the discrepancy between the 300 targeted neuropeptides and the actual 178 observed ones is due to sample biology and not a technical issue, aliquots of the four extracts were spiked with the pool containing all synthetic neuropeptides. Here, between 259 and 263 neuropeptides were detected per sample, with 259 detected in all four (∼98% sample overlap, Fig. 2*A* and supplemental Table S5). Hence, most peptides from the PRM target could successfully be detected in a neuropeptide extract with synthetic spike-ins, which means that the missing endogenous neuropeptides are most likely not present in these samples or are below detection limit. Detection of 263 neuropeptides also leaves 37 of the 300 targeted neuropeptides undetected in the spiked samples, which is probably because of the more complex sample background of a biological extract *versus* the purely synthetic sample, which may challenge the detection of some ions.

Overall, we observed 88% of the neuropeptides targeted with PRM in relevant biological matrix. Given these excellent results, together with the label-free quantification that is possible based on the more reliable MS2 signal in PRM (65), we proceeded with this method to support hypothesis building in our search for neuropeptides that may be involved in dauer-relevant behaviors. We quantified changes in neuropeptide abundance in L3 and dauer juvenile samples of *C. elegans* (Fig. 2*B*). In these samples, 164 of the 300 targeted neuropeptides proved suitable for relative quantification (see Experimental procedures section for the applied criteria for peak selection). As the vast majority of detected neuropeptides (161) were significantly more abundant in dauer juveniles, this results in a heavily skewed volcano plot (Fig. 2*B*). The only neuropeptides of the quantified set not displaying any significant change are NLP-40-2, FLP-27-2, and FLP-1-9. Next to the observed differences in neuropeptide abundancies (○ in Fig. 2*B*), we also observed 79 neuropeptides whose presence was consistently limited to dauer juveniles only, being below detection limit in L3 animals (∇ in Fig. 2*B*).

Compared with the initial technical replicates, we observed considerably more variation between the independently grown experimental L3 and dauer samples (supplemental Fig. S3). Power analysis indicates that, with the number of replicates used in this experiment and for a confidence of 95%, the statistical power is limited to fold changes of fourfold and higher supplemental Fig. S4), and the statistical power needed for the detection of more subtle fold changes will require more demanding sample collection (supplemental Fig. S4).

### Specific Neuropeptide Signaling Genes Affect Nictation Ratio of *C. elegans* Dauers

**Fig. 3:**
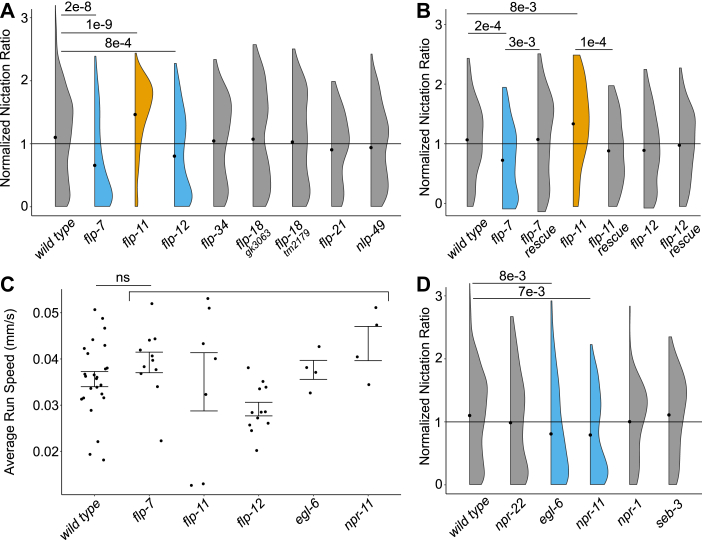
**Neuropeptides of *flp-7* and *flp-11* regulate nictation *via* (an) unknown receptor(s).***A*, Of eight prioritized neuropeptide candidates, mutants of three displayed changes in nictation ratio compared with wildtype (*p* < 0.05). Least squares means, standard error of mean, and *p* values can be found in supplemental Table S8. Corresponding initiation index and average duration: supplemental Fig. S5. *B*, restoring mutant loci to wildtype also resulted in a wildtype nictation ratio for *flp-7* and *flp-11*. The weaker *flp-12* phenotype (*A*) could not be replicated in these experiments. Significance values (*p* < 0.05) are *versus* wild type. Least squares means, standard error of mean, and *p* values can be found in supplemental Table S9. *C*, centroid tracking of neuropeptide (receptor) mutants with deviating nictation behavior. No significant changes were found compared with wildtype (*p* < 0.05). Average locomotion run speed, standard error of mean, and *p* values can be found in supplemental Table S10. *D*, of the five selected neuropeptide receptor mutants, two showed significant reduced nictation ratio compared with wildtype (*p* < 0.05). Least squares means, standard error of mean, and *p* values can be found in supplemental Table S8. Corresponding initiation index and average duration: supplemental Fig. S5. Median interexperimental normalization (*dashed line*) was applied to obtain normalized nictation ratios, *black dots* show mean values of each population. Raw data (*A* and *D*): raw_data_screen.csv, (*B*): raw_data_rescues.csv, and (*C*): raw_data_L3_centroid_tracking.csv.

To prioritize neuropeptide candidates for testing their causal involvement in nictation, we combined our discovery and differential data and filtered for neuropeptide genes that are present in *S. carpocapsae* and *C. elegans* (as a shared behavior might result from shared genetics; supplemental Tables S3 and S4) and whose peptides are more abundant in dauers *versus* L3 juveniles (Fig. 2*B*). We further prioritized neuropeptides based on reported involvement in food searching or sensing, metabolism, or insect infection (11, 66, 67, 68, 69, 70, 71). The following arguments were taken into consideration: (1) *flp-*7 and *flp-11* mRNAs are highly upregulated upon host entry in *Steinernema* spp. (67); (2) *flp-7* has been proven to mediate serotonergic body fat loss in *C. elegans via npr-22*, a luqin-like receptor (68); (3) the *flp-34*/*npr-11* NPY/NPF-like system is involved in feeding behavior in *Drosophila melanogaster* (66) as well as in olfactory learning in *C. elegans* (69); (4) *flp-12* is crucial for normal locomotion in *Globodera pallida*, a plant-parasitic nematode (71); (5) RNAi targeting of *flp-21* affects nictation in *S. carpocapsae* (11); (6) *flp-21* and *flp-18*, together with their receptor *npr-1*, have been implicated in social feeding in *C. elegans* (72); and (7) *nlp-49* mRNA is increased in *S. carpocapsae* strains that nictate less (12). Taken together, we selected seven neuropeptide-encoding genes *(flp-7*, *flp-11*, *flp-12*, *flp-18*, *flp-21*, *flp-34*, and *nlp-49*) as likely candidates to be involved in the modulation of nictation and tested possible effects of their absence, as well as that of their cognate receptors, whenever known, on *C. elegans* nictation behavior (Fig. 3*A*).

Using mutants of the aforementioned neuropeptides, we measured nictation ratios by taking the ratio of time nictating over total observed time for individual worms. We found that *flp-11* mutants have an increased nictation ratio (55 ± 3%, *p* = 1.9e-9) compared with wildtypes (37 ± 1%). Furthermore, mutants for *flp-7* (22 ± 3%, *p* = 2.7e-8) and *flp-12* (26 ± 3%, *p* = 8.4e-4) nictated less than wildtypes. Five of the tested strains—mutants for *flp-34* (36 ± 3%, *p* = 0.99), *flp-18 (gk3063)* (34 ± 3%, *p* = 0.98), *flp-18 (tm2179)* (38 ± 3%, *p* = 0.99), *flp-21* (35 ± 3%, *p* = 0.99), and *nlp-49* (33 ± 3%, *p* = 0.84)—did not significantly differ from wildtype (Fig. 3*A* and supplemental Table S8).

To confirm the suggested roles of *flp-7*, *flp-11*, and *flp-12* in nictation behavior, we relied on CRISPR–Cas9 to restore these mutated peptide genes to their wildtype alleles. Unfortunately, we were not able to replicate the phenotype of the *flp-12* mutant, questioning its role in the behavior. However, for the *flp-11* and *flp-7* endogenous rescue strains, the nictation ratio was not significantly different from wildtype, confirming causality (Fig. 3*B* and supplemental Table S9).

We hypothesized that some observed nictation defects might not be specific to this behavior but rather reflect more general problems with locomotion. To exclude this possibility, we tracked worm populations at the L3 stage and compared their crawling speed to that of wildtype controls. We found that none of the tested mutants had changed crawling speed (Fig. 3*C* and supplemental Table S10). We conclude that the nictation defects observed in the *flp-7* and *flp-11* mutants are most likely sufficiently specific to this behavior and not caused by neuromuscular defects resulting in general locomotion problems.

Next, we wanted to test the role of *npr-22*, a known neuropeptide receptor of *flp-7* and *flp-11* (73). However, *npr-22* mutants did not show a significantly different nictation ratio compared with wildtype (32 ± 3%, *p* = 0.32; Fig. 3*D*). We also probed for nictation defects of known neuropeptide receptor mutants of other neuropeptide candidates and of neuropeptides with a known role in nictation: *egl-6* (*flp-10* and *flp-17* for their implication in modulation of nictation (21)), *npr-11* (*flp-34* (66)), *npr-1* (*flp-21* (72)), and *seb-3* (*nlp-49* (74)). Both *egl-6* (23 ± 3%, *p* = 8.05e-3) and *npr-11* (28 ± 3%, *p* = 7.0e-3) mutants have a reduced nictation ratio compared with wildtype. Mutants of *npr-1* (43 ± 3%, *p* = 0.86) and *seb-3* (38 ± 3%, *p* = 0.99) do not significantly differ from wildtype (Fig. 3*D*).

## Discussion

Nictation is used by several parasitic and free-living nematodes during host finding. Research has shown that neuropeptide mRNAs are highly abundant during the IJ stage (12, 21, 23), raising the question what their function might be in the regulation of nictation. Here, we showed that *S. carpocapsae* IJs and *C. elegans* dauers contain a rich neuropeptide complement and that virtually all neuropeptides are more abundant in the dauer *versus* L3 juvenile stages in *C. elegans*. Our behavioral assays propose *flp-7* and *flp-11* as modulators of nictation.

Neuropeptide precursor proteins undergo extensive post-translational cleavages to produce the mature bioactive peptides. As a result, well-established quantitative approaches such as RNA-Seq (mRNA abundance) or proteomics (precursor protein abundance) cannot deliver quantitative insights into mature neuropeptide biology. To overcome this, we developed a PRM LC–MS/MS strategy to quantify the bulk of the *C. elegans* neuropeptidome and applied this to monitor changes in neuropeptide levels between L3 and dauer juveniles. Up until now, peptidomics workflows relied heavily on DDA, but this comes with a number of drawbacks. First, DDA selects peaks for fragmentation based on intensity. While this strategy is excellent for discovery purposes, it is inherently biased toward the high-abundant ions present in a sample, which typically does not include neuropeptides. Second, this *modus operandi* severely impacts reproducibility, since peak selection in DDA is stochastic, with different peaks being selected even when the identical sample is measured several times. The demand for LC–MS/MS strategies overcoming the low sensitivity and low reproducibility of DDA as well as enabling robust quantification of neuropeptides has been voiced recently (75). PRM itself has already been used previously for this purpose but was mostly limited to target a handful of neuropeptides (63), in contrast to the several hundred described in this study. When using our targeted neuropeptidomics method, we observed high variation between the independently grown replicates. While it should be emphasized that a high degree of scrutiny is essential during worm culturing and sample preparation to minimize technical variation, a large part is due to biology (supplemental Fig. S3, technical *versus* biological replicates). CVs at the neuropeptide level, as seen in our experiments, are typically higher than those observed in proteomics workflows (76, 77), especially for low-abundant neuropeptides (supplemental Fig. S3). As opposed to peptidomics, quantitative proteomics benefits from inferring protein abundances from multiple peptides per target protein, thereby averaging out variation observed at the peptide level. In addition, proteins tend to be more stable than neuropeptides, the latter having a higher turnover because of their signaling function (typically in the range of several minutes) (78, 79). Because of this inherently larger variation, detecting small differences in neuropeptide levels currently requires increasing the number of sampled replicates per condition. We here benefited from the very high fold changes in dauers *versus* L3s for high-confidence differential statements (Fig. 2*B*), but the detection of more subtle effect sizes (10–20% difference) with 95% confidence for the majority of neuropeptides would have required over 20 samples per condition (supplemental Fig. S4). Currently, the neuropeptidomics workflow described here makes use of whole-mount extracts, thereby erasing any spatial information. Approaches for sorting fluorescently marked *C. elegans* neurons for RNA-Seq purposes already exist (80); however, we here collected between 0.6 and 1 ml of biological material per sample for peptidomics, and approximating this using individually sorted cells is not yet practically possible. Attempts to reduce the amount of biological material would be interesting for future optimization, but there is a risk of increasing sample variation because of low abundance (supplemental Fig. S3).

Our research strategy was based on the common neuropeptide complement between *C. elegans* and *S. carpocapsae* IJs and on the increased abundance of neuropeptides in dauers compared with L3 juveniles. Although the first criterion reduced the target list to 45 neuropeptide genes (45% of the known *S. carpocapsae* complement), the differential abundance criterion only provided very minimal prioritization, as only two of the three neuropeptides that were not significantly more abundant were present in the pool of 45 genes (reducing the target list to 43 genes). Additional strategies to narrow down our target list were therefore based on forward genetic screens (27) or RNA-Seq (12, 21). The impressively high abundance of neuropeptides in dauer juveniles overall is quite intriguing and can be due to substantial differences in neuropeptide storage and/or release and/or turnover. One possibility is that dauer juveniles may be massively translating neuropeptides from the already highly abundant mRNA (21), and stocking these, but the rather high mRNA levels, do not support this very well and rather support high turnover. A second possibility is for all these neuropeptides to contribute to the execution of dauer behaviors and physiological functions, which is partially supported by our findings that *flp-7* and *flp-11* are involved in mediating nictation, a dauer-specific behavior. It is not known whether these neuropeptides are also more abundant when regulating their other known roles (68, 81, 82, 83, 84) or even whether there would be a general correlation between neuropeptide abundance and usage. It remains curious that almost all quantified neuropeptides are so plentiful, as it seems unlikely that dauers need all of them simultaneously. Although generally, transcription correlates poorly with translation (85, 86, 87), neuropeptide mRNA (21) and actual neuropeptide levels (this study) are both convincingly increased in dauers compared with L3/L4 juveniles (supplemental Table S11). While there are several exceptions to this general correlation, it suggests that many neuropeptide genes may be actively transcribed *and* translated in the dauer stage. Having developed quantitative peptidomics, these and other questions are now within reach of addressing in future studies.

IJs and dauers alternate nictation bouts with crawling. Animals may therefore display different nictation ratios because of differences in duration of nictation bouts and/or because of increased or decreased occurrence of these bouts. For example, our data suggest that *flp-11* mutants spend more time nictating because of nictation stamina (longer bouts, Figs. 3, *A*, *B*, S5*B* and S6*B*). We also noticed that mutants with a decreased nictation ratio nearly always had a reduced initiation index (except for *egl-6* mutants; Figs. 3, S5*A* and S6*A*). We hypothesize that in these mutants, a reduced or lack of nictation initiation signal may be causal to the observed decrease in nictation ratio. However, with low numbers of strains evaluated and *egl-6* being an exception, we would urge readers to currently refrain from generalizing these observations. The observations of changed initiation index and average duration of nictation would have remained unnoticed without the use of individual nictation assessment. This shows that nictation ratio on its own is not a particularly sensitive readout, and there is merit in measuring more subtle parameters when aiming to unveil genetic contributors to this behavior.

Because neuropeptides require receptors to propagate their signal, one might hope for known receptor–ligand couples to display similar nictation phenotypes. However, we did not observe matching nictation effects for the NPF(R)-like (69) *flp-34* and *npr-11* genes (Fig. 3*D*) or for the tachykinin(-receptor)-like (68, 73) *flp-7* and *npr-22* genes (Fig. 3, *A* and *D*); suggesting that (an)other neuropeptide ligand(s) may be relevant here (88, 89, 90). Several neuropeptide receptors are known for their promiscuous interactions with ligands (88, 91); hence, specific behaviors might be modulated *via* differentiation of ligand–receptor couple use (92).

We found *flp-21* and its receptor *npr-1* to both not influence nictation ratio (although the latter did display longer nictation bouts; supplemental Fig. S5 and supplemental Table S8). This was surprising since Dalzell *et al.* showed that *flp-21*-targeted RNAi reduces nictation in *S. carpocapsae*. This apparent lack of conserved role raises the question whether *C. elegans* is a good general model for nictation (or even other behaviors) of other nematodes. However, we cannot yet rule out technical differences as basis for these different observations, since targeted mutation (*C. elegans*) is very different from RNAi (*S. carpocapsae*). As more tools are being developed for more species, it will be interesting to use these to probe for functional evolution and diversification of neuropeptide signaling systems over nematodes.

This study provides a comprehensive library of *S. carpocapsae* IJs and *C. elegans* neuropeptides and delivers a differential neuropeptidomics approach for their quantification. Of the here identified neuropeptide genes, 20 (*nlp-99* through *nlp-118*) are novel to the nematode phylum, bringing the total to 118 neuropeptide-like proteins (*nlp*). On top of the 56 known *S. carpocapsae* neuropeptide genes, we were able to identify 41 genes that were unannotated before. Of the 20 that are novel to the nematode phylum, 19 do not seem to have an ortholog in the *C. elegans* genome. While we here focused on the conserved genes, the 19 *S. carpocapsae* genes might be related to parasitic life styles (supplemental Table S7). These genes may therefore represent interesting targets for research into parasitism, with possible applications relevant to human health or agriculture.

We set out to find molecular regulators of nictation because of its role in the host-finding cycle of some EPNs (59, 93). Understanding how nictation is regulated is important because it might increase the application potential of EPNs as biopesticides for specific insect pests (93); however, it is not yet known whether an EPN with an optimized nictation ratio for a specific pest is more effective in the field. These data advance our understanding of the neuropeptidergic complement of nematodes, provide fundaments to untangle how nictation behavior is regulated, and open the door for comparative peptidomics in many other areas of research.

## Data Availability

All MS data pertaining neuropeptide discovery in *S. carpocapsae* have been deposited to the ProteomeXchange Consortium *via* the PRIDE (94) partner repository with dataset identifier PXD034629 and 10.6019/PXD034629. The Skyline analysis files of the targeted MS experiments in *C. elegans* L3 and dauers have been deposited to Panorama Public (95) and can be accessed *via* https://panoramaweb.org/targeted_neuropeptidomics-L3_vs_dauer.url.

## Supplemental data

This article contains supplemental data.

## Conflict of interest

The authors declare no competing interests.
