# Supplementary material for: Mass Spectrometry–Driven Discovery of Neuropeptides Mediating Nictation Behavior of Nematodes
Source: Mol Cell Proteomics. 2022 Dec 5;22(2):100479. doi: 10.1016/j.mcpro.2022.100479 (PMC9881375; doi:10.1016/j.mcpro.2022.100479)
Supplement: Supplemental data [file mmc1.docx]

# Supplemental data

Mass spectrometry-driven discovery of neuropeptides mediating nictation behavior of nematodes

**Bram Cockx^[1]^*, Sven Van Bael^[1]^*, Rose Boelen^[1]^, Elke Vandewyer^[1]^, Heeseung Yang^[4]^, Tuan Anh Le^[1]^, Johnathan J Dalzell^[3]^, Isabel Beets^[1]^, Christina Ludwig^[2]^, Junho Lee^[4]^, Liesbet Temmerman^[1]^**

[1] Animal Physiology & Neurobiology, Department of Biology, University of Leuven (KU Leuven), Leuven, Belgium

[2] Bavarian Center for Biomolecular Mass Spectrometry (BayBioMS), Technical University of Munich (TUM), Freising, Germany.

[3] School of Biological Sciences, Queen’s University Belfast, Northern Ireland, United Kingdom.

[4] Department of Biological Sciences, Seoul National University, Seoul, South Korea.

*These authors contributed equally to this work.

## Supplemental tables

**Table S1: strains used in this work (Excel file).**

**Table S2: Neuropeptide results of data-dependent acquisition LC-MS/MS in *S. carpocapsae* (Excel file).** Columns A and B contain the names of the precursor and individual neuropeptide sequences. Columns C and D show the detected neuropeptide sequences, together with the observed modification mass shifts (between brackets, and further specified in column K). Columns E to I show the -lg10P value and the observed m/z, charge (z), mass and mass error (in ppm). Column J lists the individual raw files in which that particular sequence was detected. All raw files are available via the PRIDE repository that accompanies this publication.

**Table S3: Known, predicted and new neuropeptide precursors of S. carpocapsae (Excel file)**. Columns A‑F contain information on the precursor level: assigned or newly proposed name, gene ID, color code referring to the groups in Figure 1, and origin of in silico prediction data). Columns G-I contain information on the mature peptides, with proposed names (whenever possible, matching the corresponding peptide name in C. elegans based on location in the precursor), the peptide sequence and supporting evidence for this sequence.

**Table S4: Neuropeptide gene orthology in *C. elegans* and *S. carpocapsae* (Excel file).** Overview of neuropeptide proteins that are conserved between *C.* *elegans* and *S. carpocapsae*. A black box indicates that there is evidence (literature, MS, or *in silico*) for that specific neuropeptide in the species.

**Table S5: Neuropeptides detected using parallel reaction monitoring in four *C. elegans* technical replicates (Excel file).** Overview of all neuropeptide sequences detected in four extracts with or without addition of the synthetic neuropeptide pool. Detected sequences are indicated in red for each sample.

**Table S6: Neuropeptides detected using data-dependent acquisition in four *C. elegans* technical replicates (Excel file).** Overview of all neuropeptide sequences detected in four extracts with or without addition of the synthetic neuropeptide pool. Detected sequences are indicated in red for each sample.

**Table S7. Newly discovered neuropeptide precursors in *S. carpocapsae* (Excel file).** Precursor sequences were compared to *C. elegans*, other *Steinernema* spp. and other nematodes using BLASTp (Camacho et al., 2009). All newly found precursors had a sequence homolog in at least one other Steinernema spp. and in seven out of the 21 cases, homologs were also found in other nematode species. For *nlp-99* we also found an orthologs in *C. elegans* which has a neuropeptide-like profile (signal peptide and conserved dibasic cleavage sites).

**Table S8. L.S.M., S.E.M. and p-values of nictation ratio, initiation index and average duration of selected candidates (Excel file).** Raw data can be found in the supplemental data file raw_data_screen.csv.

**Table S9. L.S.M., S.E.M. and p-values of nictation ratio, initiation index and average duration of CRISPR-Cas9 rescue strains (Excel file).** Raw data can be found in the supplemental data file raw_data_rescues.csv.

**Table S10. L.S.M., S.E.M. and p-values of the locomotion run speed of L3 worms (Excel file).** Only mutants from the mutant screen with deviating nictation ratio were tested for changes in locomotion. Raw data can be found in the supplemental data file raw_data_L3_centroid_tracking.csv .

**Table S11. Overview of neuropeptides that are more or less abundant on mRNA and peptide level (Excel file).** mRNA data originated from Lee *et al.* 2017 (J. S. Lee et al., 2017) and compared qualitatively to our own data. mRNA and peptide abundances correlate well.

## Supplemental figures


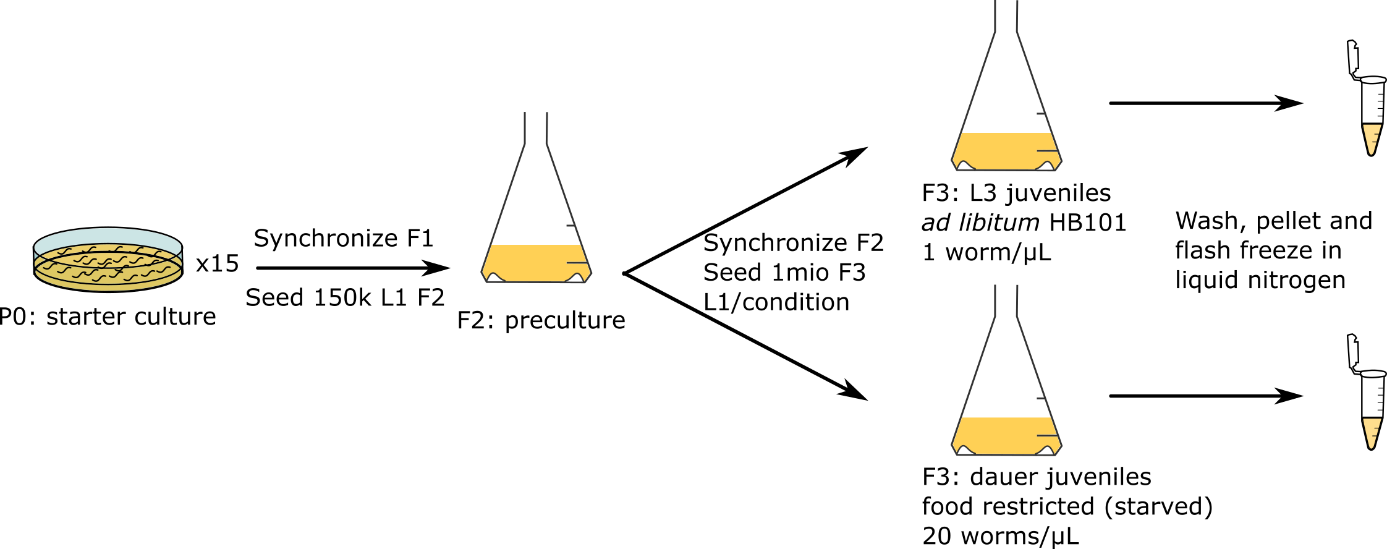


**Figure S1. Overview of sampling strategy for C.** **elegans dauers used for peptide extractions.** A starter culture of 15 plates with each 10 young adults is grown for 4 days (96 hours) and synchronized. A 150k L1 worms are seeded as a preculture. 72h later they are synchronized again, and the population is split into an *ad libitum* / non-overcrowded culture (sampled at L3 stage, 34h after seeding) and into a food restricted / overcrowded culture (sampled at dauer stage, 72h after seeding).

**Figure S2. *C.*** ***elegans* neuropeptide precursor alignments show conserved mature peptide sequences
in *S.*** ***carpocapsae* (PDF file).** Sequence homologs were identified using BLASTp (Camacho et al., 2009) and aligned using Clustal Omega (Sievers and Higgins, 2018). Gray-scale coloring is used to indicate conservation, from not conserved (white) to identical (dark-gray). Red rectangles were drawn over conserved known *C.* *elegans* mature peptide sequences. Bold sequences were detected using mass spectrometry (MS2 accuracy).

**
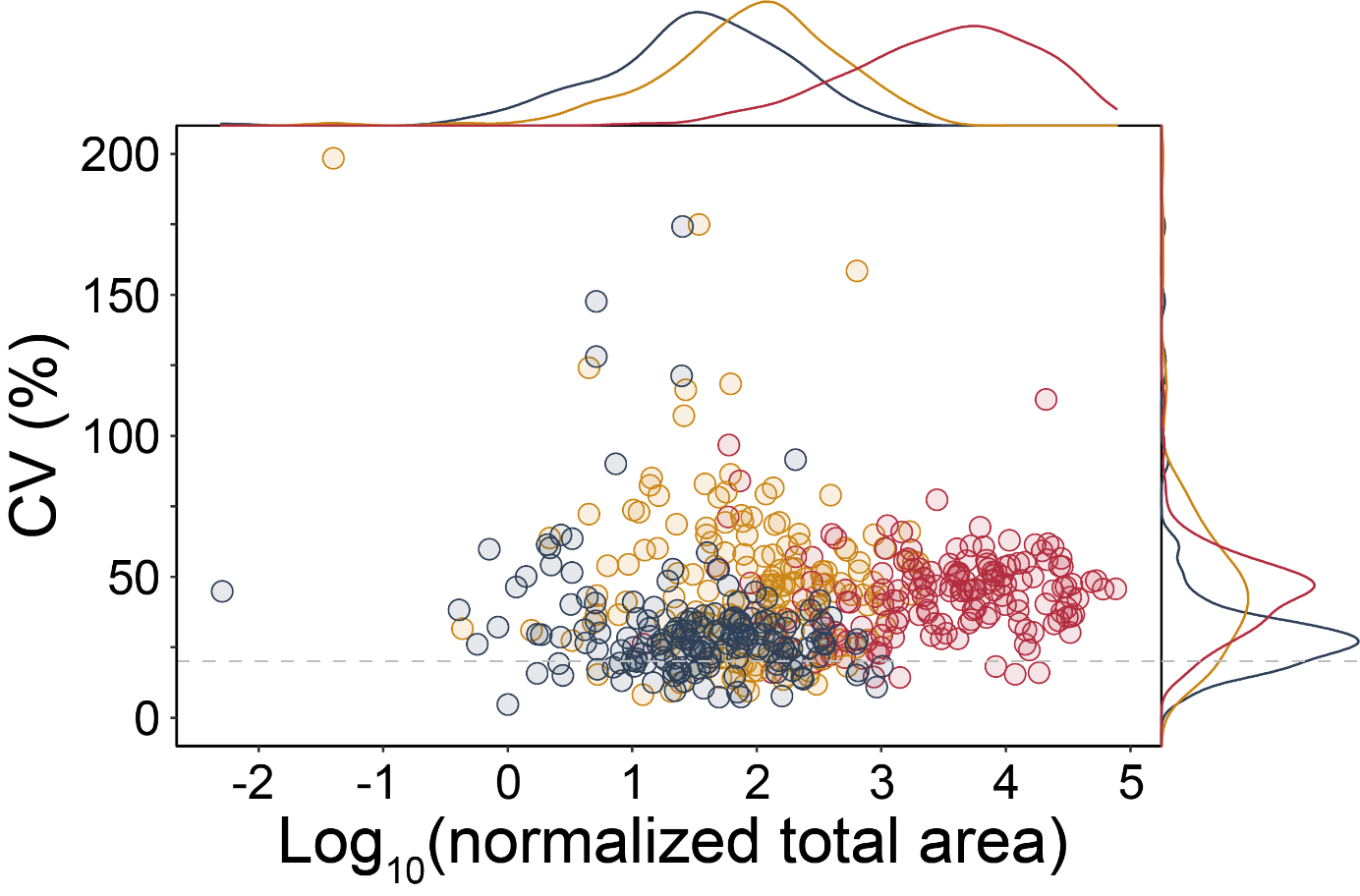
**

**Figure S3. Coefficients of variation and area under the curve of neuropeptides detected in the four mixed-stage technical replicates (black), L3 (yellow) and dauer (red) juveniles.** The gray line indicates a CV of 20%. Variation is affected by sample collection, as exemplified by the technical replicates, which display the least variation. This effect is aggravated when neuropeptides are present in low amounts, as seen in the L3 juveniles, where CV values are much broader when compared to both the technical replicates and dauer juveniles.


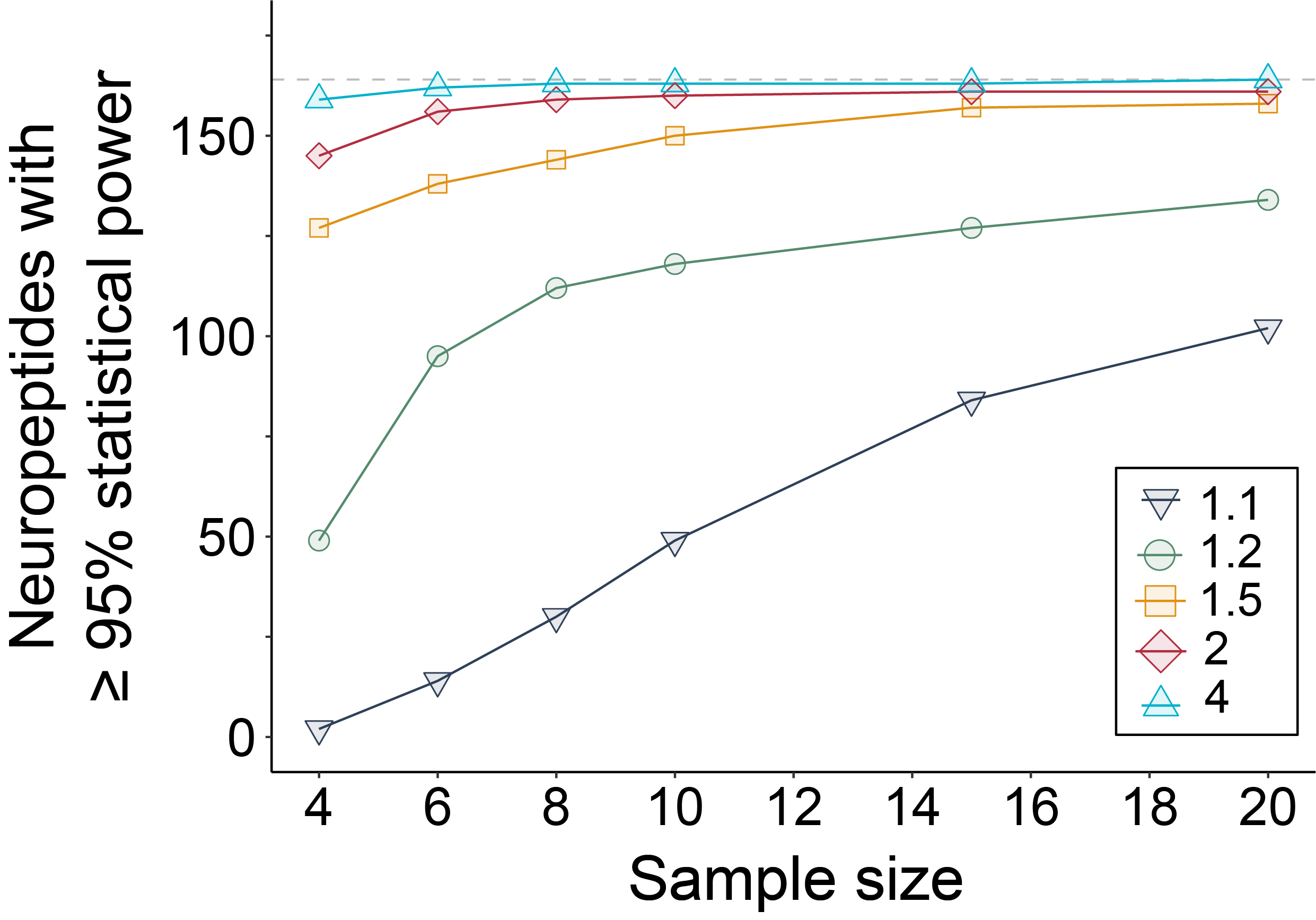


**Figure S4. Statistical power of the targeted PRM method.** Using the statistics of the L3/dauer experiment, data was simulated for different sample sizes (4, 6, 8, 10, 15 and 20 replicates, shown on the x-axis) and fold changes (1.1, 1.2, 1.5, 2 and 4, indicated with shaded shapes). Shown on the y-axis are the number of neuropeptides (out of a total of 164 that were experimentally observed) for which a Student *t*-test resulted in a p-value lower than 0.05 in at least 95% of the simulated data (500 iterations for each parameter). The analysis confirms what can be seen in the actual data, *i.e.* our statistical power is maximal for fold changes of 4 and higher. For the statistically significant detection of more subtle changes, more replicates will be required. For example, the number of replicates required for observing 1.1 and 1.2 fold changes with high statistical power exceeds 20.


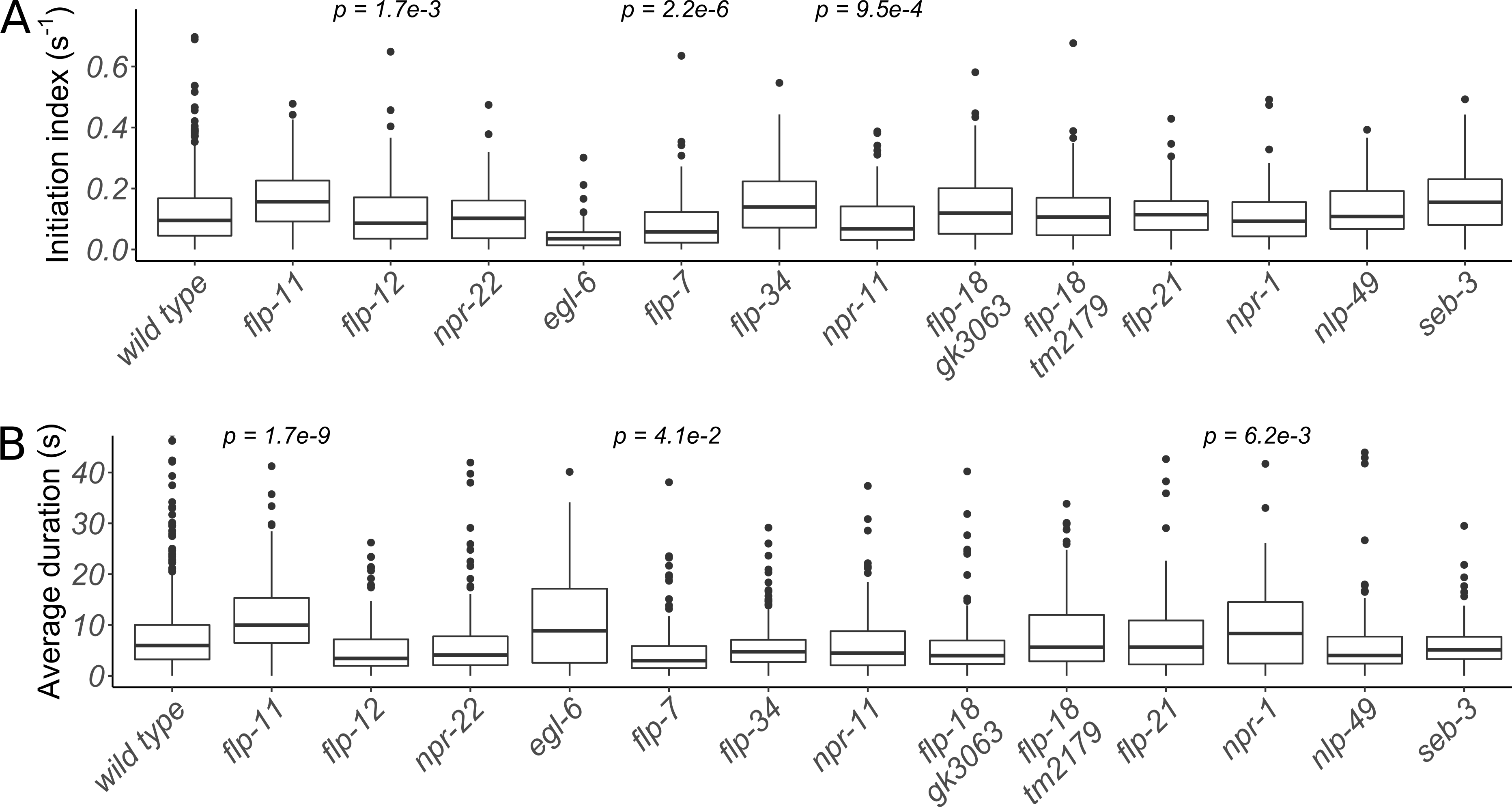


**Figure S5.** **Initiation index and average duration of prioritized candidates (Fig.** **3A).** **(A)** Initiation indices of flp-12, dmsr-8, flp-7 and npr-11 mutants are significantly lower compared to wild type worms. **(B)** Average duration of nictation bouts of flp-11 and npr-1 mutants is significantly longer than that of wild type worms while those of frpr-8 and flp-7 mutants is shorter.


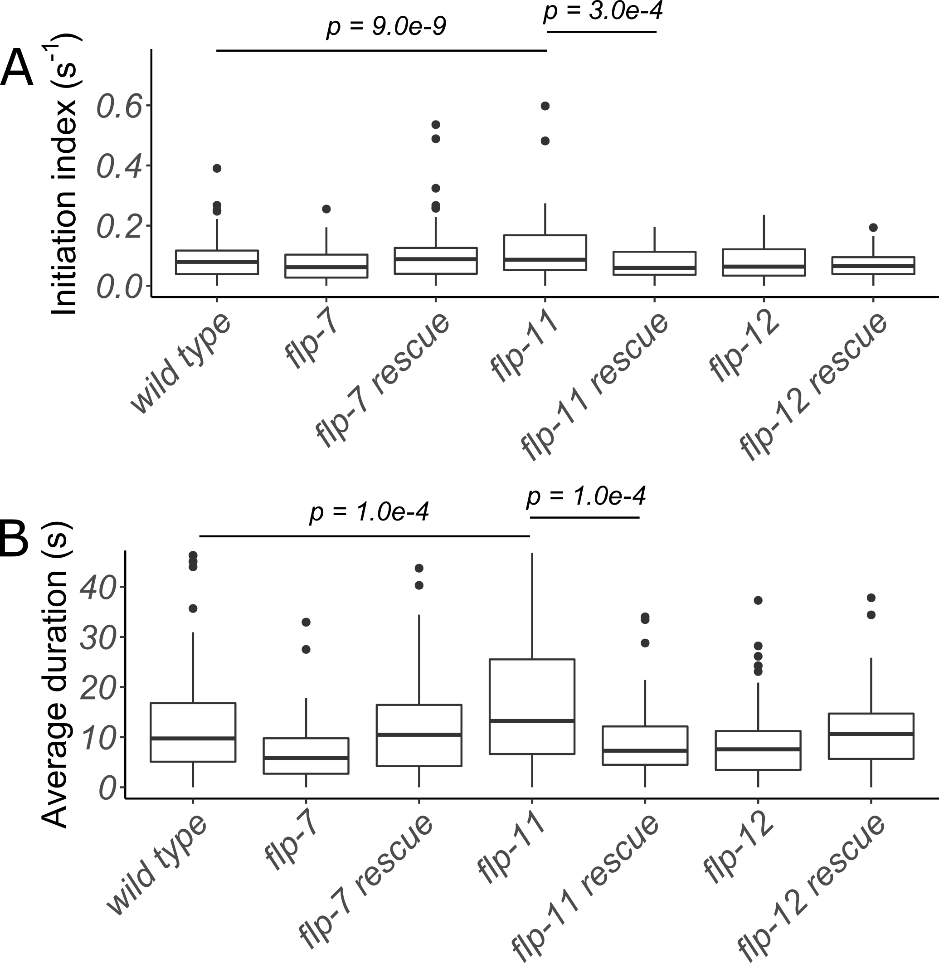


**Figure S6. The nictation initiation index and average duration of the rescue strains.** **(A)** Initiation index of flp-11 mutants is significantly higher than that of wild type worms. This phenotype is rescued when the original flp-11 allele is restored. **(B)** The average duration of flp-11 mutants is significantly longer than that of wild type worms and is again rescued when the wild-type gene is restored.
