# Supplementary material for: Mass Spectrometry–Driven Discovery of Neuropeptides Mediating Nictation Behavior of Nematodes
Source: Mol Cell Proteomics. 2022 Dec 5;22(2):100479. doi: 10.1016/j.mcpro.2022.100479 (PMC9881375; doi:10.1016/j.mcpro.2022.100479)
Supplement: Supplemental data 4 [file mmc6.pdf]

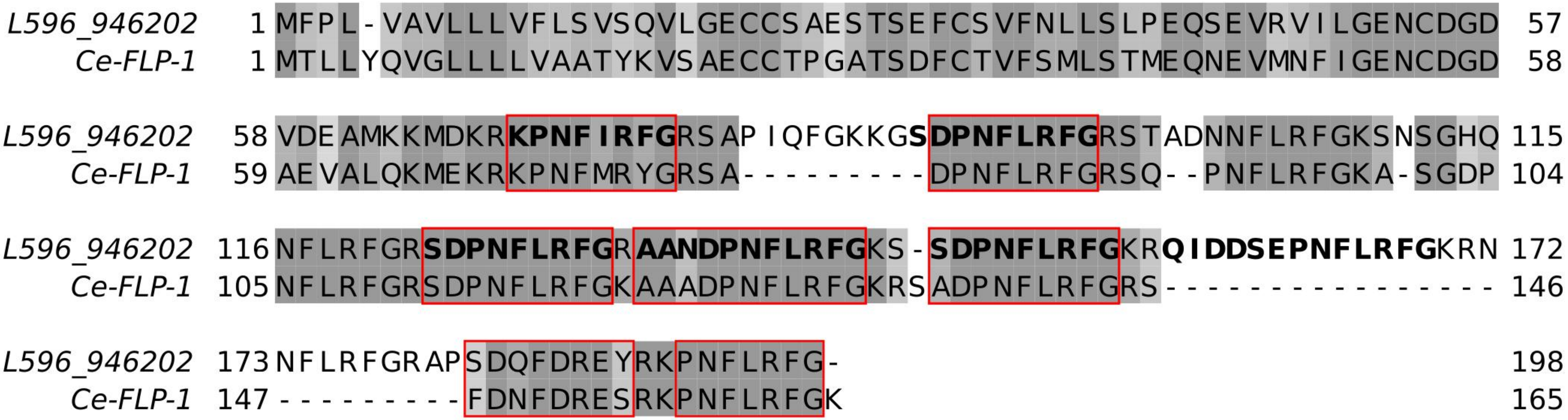
L596_946202 1 MFPL-VAVLLLVFLSVSQVLGECCSAESTSEFCSVFNLLSLPEQSEVRVILGENCDGD 57
Ce-FLP-1 1 MTLLYQVGLLLLVAATYKVSAECCTPGATSDFCTVFSMLSTMEQNEVMNFIGENCDGD 58
L596_946202 58 VDEAMKKMDKRKPNFIRFGRSAPIQFGKKGSDPNFLRFGRSTADNNFLRFGKSNSGHQ 115
Ce-FLP-1 59 AEVALQKMEKRKPNFMRYGRSA---------DPNFLRFGRSQ--PNFLRFGKA-SGDP 104
L596_946202 116 NFLRFGRSDPNFLRFGRAANDPNFLRFGKS-SDPNFLRFGKRQIDDSEPNFLRFGKRN 172
Ce-FLP-1 105 NFLRFGRSDPNFLRFGKAAADPNFLRFGKRSADPNFLRFGRS---------------- 146
L596_946202 173 NFLRFGRAPSDQFDREYRKPNFLRFG- 198
Ce-FLP-1 147 ---------FDNFDRESRKPNFLRFGK 165

```
L596_944516   1 MAAYSSIPVQSHMSSSTLSYLFFLLLLVAVFQTSSAYEEKSDYDMAEMTPYELQVKRF  58
  Ce-FLP-2    1 -----------MQVSGILSALFLVLL--AVIGTTVAQPAVNDNT-LGIFEASAMAKRL  44

L596_944516  59 RTEPIRFGKRGPREPIRFGKRAMTNSYAPHRLPGFPAFYGTYNH               102
  Ce-FLP-2   45 RGEPIRFGKRSPREPIRFGKRFNPLPDYDFQ-------------                76
```

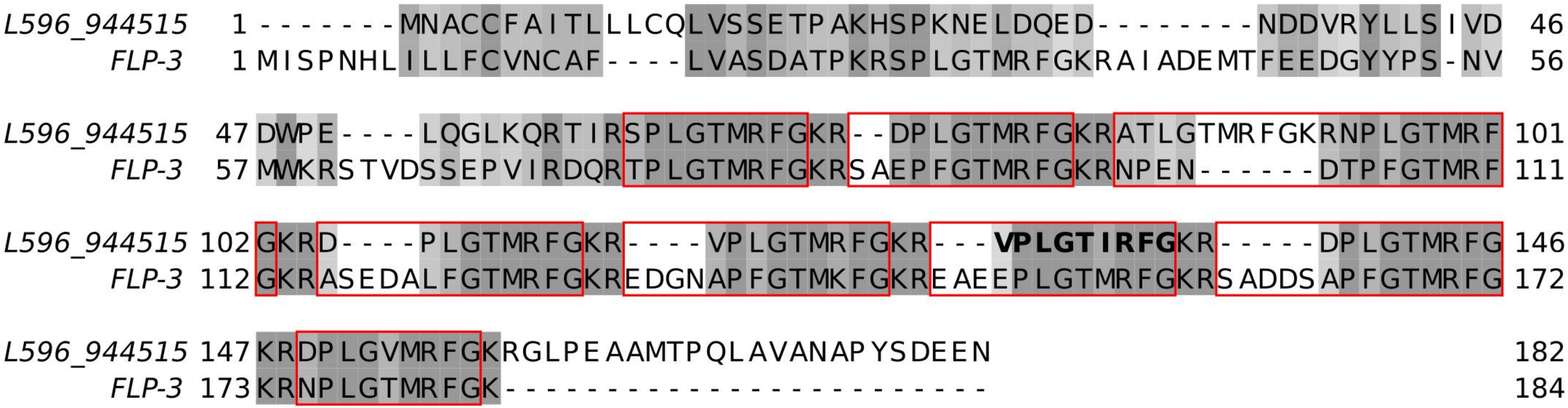
L596_944515 1 -------MNACCFAITLLLCQLVSSETPAKHSPKNELDQED--------NDDVRYLLSIVD 46
FLP-3 1 MISPNHLILLFCVNCAF----LVASDATPKRSPLGTMRFGKRAIADEMTFEEDGYYPS-NV 56
L596_944515 47 DWPE----LQGLKQRTIRSPLGTMRFGKR--DPLGTMRFGKRATLGTMRFGKRNPLGTMRF 101
FLP-3 57 MWKRSTVDSSEPVIRDQRTPLGTMRFGKRSAEPFGTMRFGKRNPEN------DTPFGTMRF 111
L596_944515 102 GKRD----PLGTMRFGKR----VPLGTMRFGKR---**VPLGTIRFG**KR-----DPLGTMRFG 146
FLP-3 112 GKRASEDALFGTMRFGKREDGNAPFGTMKFGKREAEEPLGTMRFGKRSADDSAPFGTMRFG 172
L596_944515 147 KRDPLGVMRFGKRGLPEAAMTPQLAVANAPYSDEEN 182
FLP-3 173 KRNPLGTMRFGK------------------------ 184

```
L596_941121  1 --MYVRMLNTIV---LFLIGLVLVARCAPVAEENDLFDQ----LYKRDQLLRSIYQLRA 50
   Ce-FLP-4  1 MNAFSSSLKTFIFSLLFATLLALTAA-HPPSSGEEIAEQEEKNIASPDELIPEIVEQQN 58

L596_941121 51 DSPYVSSFMRASRSNGKPTFIRFGKRSGQPYPAVYDSRA                     89
   Ce-FLP-4 59 FWPP-VHLRGLRSSNGKPTFIRFGKRASPSFIRFGK---                     94
```

```
L596_029879   1 MSASWIRILLFFVVALVAV----------QEAVGKNMERDARAPKPKFIRFGRGGGGQ  48
   Ce-FLP-5   1 MSSRSTTIAFLFIATLLVFQCVSAQSSAEDADYLEKYQRIARAPKPKFIRFGRAG--A  56

L596_029879  49 KFIRFGRSGAARQVLNPQDIYANQFYDPLEVDSAASFLDSAALAAPANDWQQFKRGGP 106
   Ce-FLP-5  57 KFIRFGRSRNTWED-----GYASPS-----------------------VNELYVKRGA  86

L596_029879 107 KFIRFG                                                     112
   Ce-FLP-5  87 KFIRFG                                                      93
```

```
L596_027509   1 MRPLCLAVTVSFLAIASGFKPVQRPLAIEGGATSSLDDGLFCENFPKHPACEAQVGQQ  58
Ce-FLP-6      1 MN--------------------SRGLIL--------TLGVVI--AVAFAQQDSEVERE  28

L596_027509  59 MEKRKSAYMRFGRSDGGL--DMEKRKSAYMRFGKRGADEEVF----EAEEPMEKRKSA 110
Ce-FLP-6     29 MMKRKSAYMRFGRSDGGNPMEMEKRKSAYMRFGKRSSGGDEQELVGGDDIDMEKRKSA  86

L596_027509 111 YMRFGKRKSAYMRFGKRGDATFDDDSMAMEKRKSAYMRFGKRSDNVEAMS-------- 160
Ce-FLP-6     87 YMRFGKR----------SGP--QEDDMPMEKRKSAYMRFGKRSSDMEVIGNEGVDGDA 132

L596_027509 161 -----------------------PFEIEKRKSAYMRFGR                    176
Ce-FLP-6    133 HDLFKRKSAYMRFGKRSMGEEEDHDMMKRKSAYMRFGR                     171
```

Ce-FLP-7	1	MLGSRFLLLALGLLVLVLAEESAEQQVQEPTELEKSGEQLSEEDLIDEQKRTPMQ	55
L596_944230	1	------MFLAYGLLLLSSFY------------LLVSGF--VLEEGMINEKRAPMD	35
Ce-FLP-7	56	RSSMVRFGRSPMQRSSMVRFGKRSPMQRSSMVRFGKRSPMQRSSMVRFGKRSPME	110
L596_944230	36	RSSMVRFGRAPMDRSSMVRFGKRAPMDRSSMVRFGKRAPMDRSSMVRFGKRAPMD	90
Ce-FLP-7	111	RSAMVRFGR-SPMDRSKMVRFGRSSIDRASMVRLGKRTPMQRSSMVRFGKRSMEF	164
L596_944230	91	RSAMVRFGKRAPMDRSSMVRFGKRTSDVQSEK----------------------	122
Ce-FLP-7	165	EMQSNEKNIEDSE	178
L596_944230		-------------	

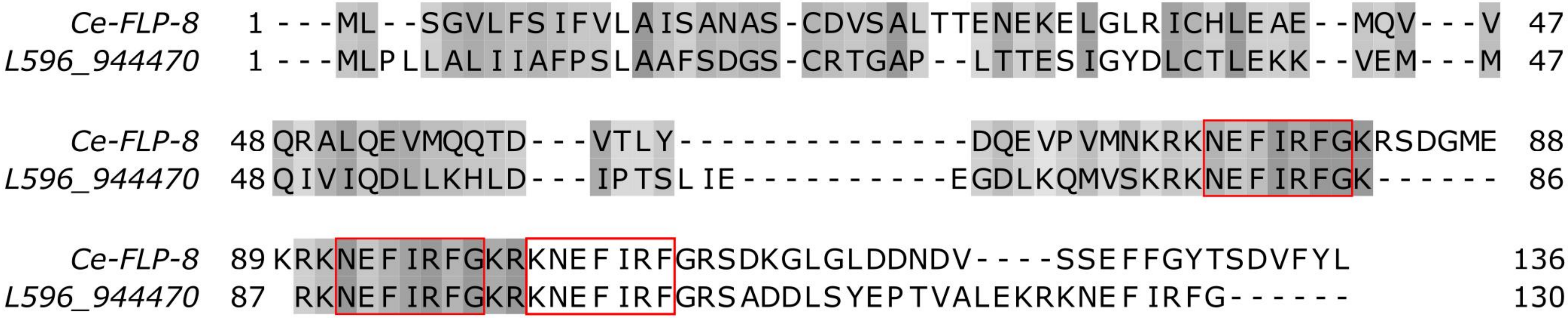

Ce-FLP-8 1 ---ML--SGVLFSIFVLAISANAS-CDVSALTTENEKELGLRICHLEAE--MQV---V 47
L596_944470 1 ---MLPLLALIIAFPSLAAFSDGS-CRTGAP--LTTESIGYDLCTLEKK--VEM---M 47
Ce-FLP-8 48 QRALQEVMQQTD---VTLY--------------DQEVPVMNKRKNEFIRFGKRSDGME 88
L596_944470 48 QIVIQDLLKHLD---IPTSLIE-----------EGDLKQMVSKRKNEFIRFGK------ 86
Ce-FLP-8 89 KRKNEFIRFGKRKNEFIRFGRSDKGLGLDDNDV----SSEFFGYTSDVFYL 136
L596_944470 87 RKNEFIRFGKRKNEFIRFGRSADDLSYEPTVALEKRKNEFIRFG------ 130

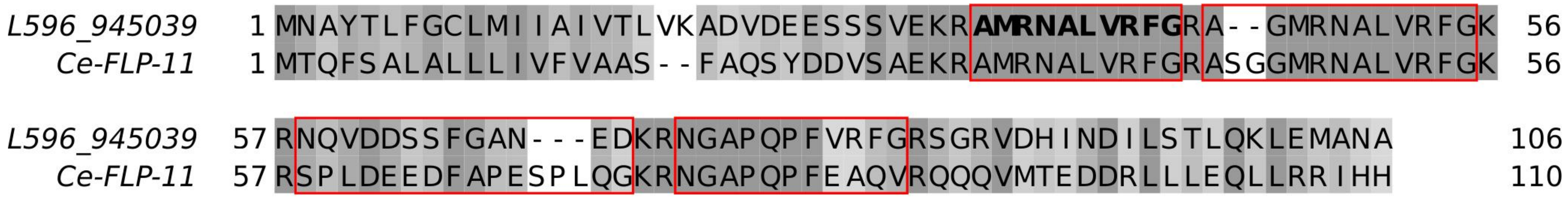

L596_945039 1 MNAYTLFGCLMIIAIVTLVKADVDEESSSVEKRAMRNALVRFGRA--GMRNALVRFGK 56
Ce-FLP-11 1 MTQFSALALLLIVFVAAS--FAQSYDDVSAEKRAMRNALVRFGRASGGMRNALVRFGK 56
L596_945039 57 RNQVDDSSFGAN---EDKRNGAPQPFVRFGRSGRVDHINDILSTLQKLEMANA 106
Ce-FLP-11 57 RSPLDEEDFAPESPLQGKRNGAPQPFEAQVRQQQVMTEDDRLLLEQLLRRIHH 110

L596_944471 1 --------------MTKGHLQSKGSPEMIDPAMVYGSD-GEMITRVQSQLLGALQMLQA 44
Ce-FLP-12 1 MNVQVIIALLFCLIATCATQKVKGSPEVLPAAMYDGELSHESVNKISAQLLNALSELEA 59

L596_944471 45 YQDGP--TKFAEKRR**NKFEFIRFG**RK 68
Ce-FLP-12 60 LQEGNQQLKMAEKRRNKFEFIRFGRK 86

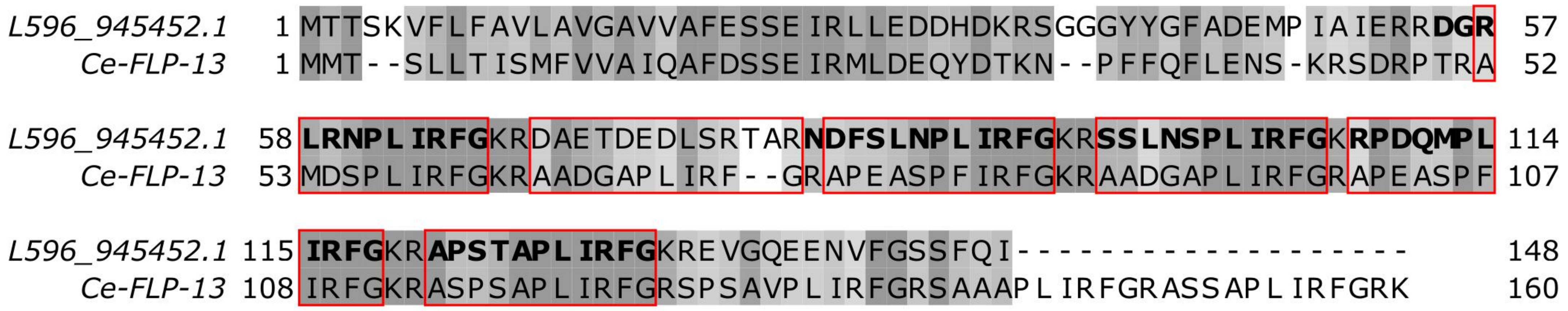

L596_945452.1 1 MTTSKVFLFAVLAVGAVVAFESSEIRLLEDDHDKRSGGGYYGFADEMP IAIERRDGR 57
Ce-FLP-13 1 MMT--SLLTISMFVVAIQAFDSSEIRMLDEQYDTKN--PFFQFLENS-KRSDRPTRA 52
L596_945452.1 58 LRNPLIRFGKRDAETDEDLSRTARNDFSLNPLIRFGKRSSLNSPLIRFGKRPDQMPL 114
Ce-FLP-13 53 MDSPLIRFGKRAADGAPLIRF--GRAPEASPFIRFGKRAADGAPLIRFGRAPEASPF 107
L596_945452.1 115 IRFGKRAPSTAPLIRFGKREVGQEENVFGSSFQI------------------- 148
Ce-FLP-13 108 IRFGKRASPSAPLIRFGRSPSAVPLIRFGRSAAAPLIRFGRASSAPLIRFGRK 160

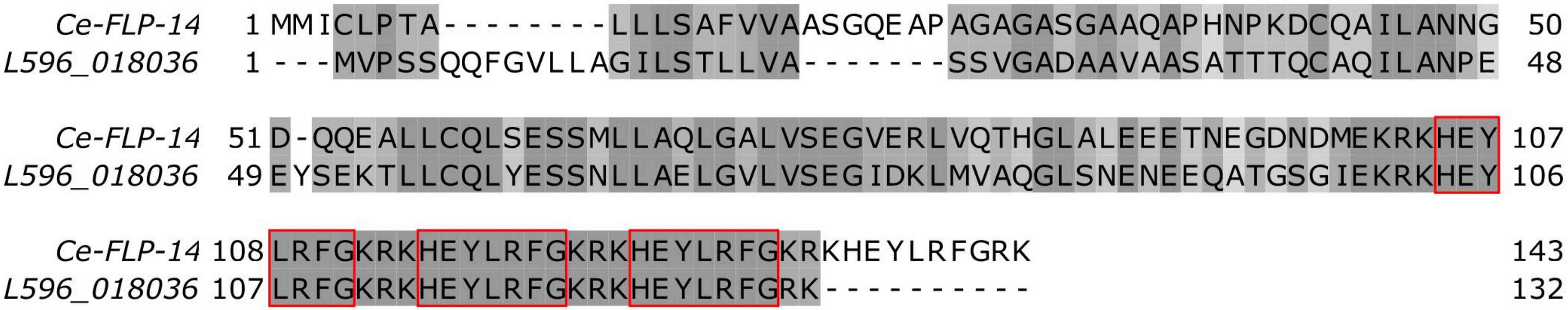
Ce-FLP-14 1 MMICLPTA--------LLLSAFVVAASGQEAPAGAGASGAAQAPHNPKDCQAILANNG 50
L596_018036 1 ---MVPSSQQFGVLLAGILSTLLVA-------SSVGADAAVAASATTTQCAQILANPE 48
Ce-FLP-14 51 D-QQEALLCQLSESSMLLAQLGALVSEGVERLVQTHGLALEEETNEGDNDMEKRKHEY 107
L596_018036 49 EYSEKTLLCQLYESSNLLAELGVLVSEGIDKLMVAQGLSNENEEQATGSGIEKRKHEY 106
Ce-FLP-14 108 LRFGKRKHEYLRFGKRKHEYLRFGKRKHEYLRFGRK 143
L596_018036 107 LRFGKRKHEYLRFGKRKHEYLRFGRK---------- 132

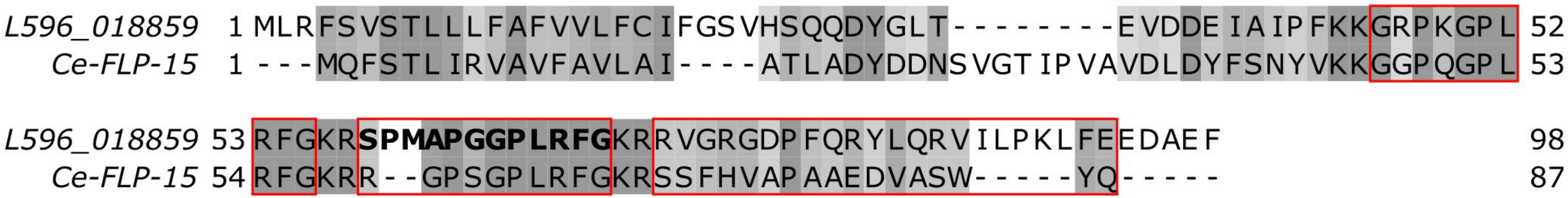

L596_018859 1 MLRFSVSTLLLFAFVVLFCIFGSVHSQQDYGLT--------EVDDEIAIPFKKGRPKGPL 52
Ce-FLP-15 1 ---MQFSTLIRVAVFAVLAI----ATLADYDDNSVGTIPVAVDLDYFSNYVKKGGPQGPL 53
L596_018859 53 RFGKRSPMAPGGPLRFGKRRVGRGDPFQRYLQRVILPKLFEEDAEF 98
Ce-FLP-15 54 RFGKRR--GPSGPLRFGKRSSFHVAPAAEDVASW-----YQ----- 87

L596_947610 1 MNVLIRIALVFISLSHVVFSSTIRNSMNQPNVA-VAAANRELERLPSQEELLNQELQN 57
Ce-FLP-16 1 -----------MNFSGFEFSSI----------------------------------VA 13

L596_947610 58 IYLAELMKQYAAENGVNTNQVEEPVNEQQAYGGMDKR**AQTFVRFG**RSSSVGASSVEEP 115
Ce-FLP-16 14 FFLLIL--QLS------T---AAVLPADYAYGVADEMS----ALPDSGSLF-AEQRPS 55

L596_947610 116 VSQQQYGGMDKR**AQTFVRFG**KR**GQTFIRFG**------- 145
Ce-FLP-16 56 KRAQTFVRFGKRAQTFVRFGKRGQTFVRFGRSAPFEQ 92

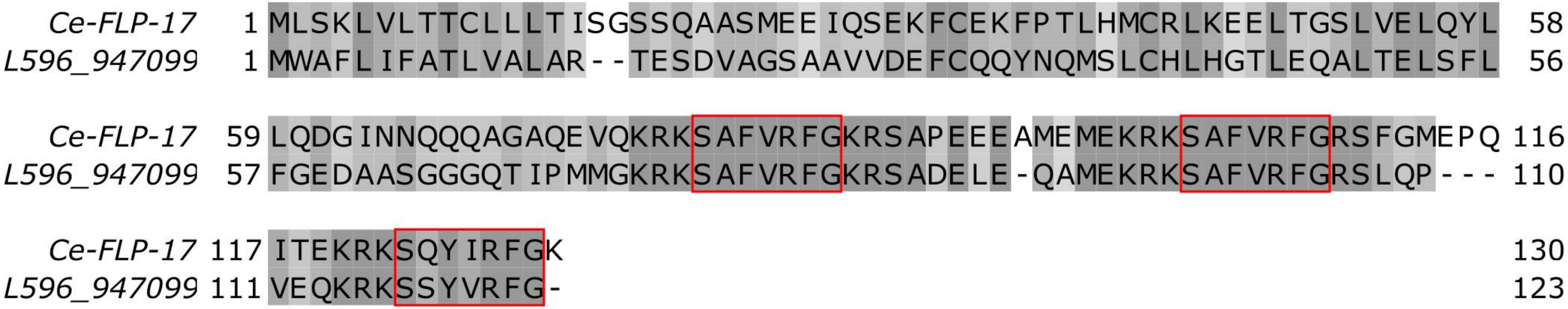
Ce-FLP-17 1 MLSKLVLTTCLLLTISGSSQAASMEEIQSEKFCEKFPTLHMCRLKEELTGSLVELQYL 58
L596_947099 1 MWAFLIFATLVALAR--TESDVAGSAAVVDEFCQQYNQMSLCHLHGTLEQALTELSFL 56
Ce-FLP-17 59 LQDGINNQQQAGAQEVQKRKSAFVRFGKRSAPEEEAMEMEKRKSAFVRFGRSFGMEPQ 116
L596_947099 57 FGEDAASGGGQTIPMMGKRKSAFVRFGKRSADELE-QAMEKRKSAFVRFGRSLQP--- 110
Ce-FLP-17 117 ITEKRKSQYIRFGK 130
L596_947099 111 VEQKRKSSYVRFG- 123

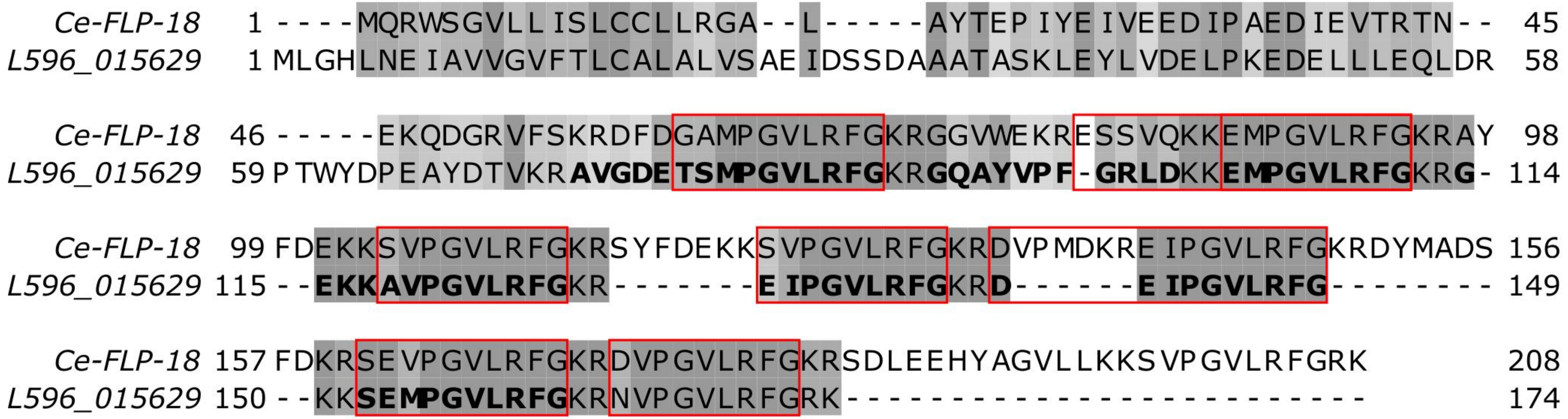
Ce-FLP-18 1 ----MQRWSGVLLISLCCLLRGA--L-----AYTEPIYEIVEEDIPAEDIEVTRTN-- 45
L596_015629 1 MLGHLNEIAVVGVFTLCALALVSAEIDSSDAAATASKLEYLVDELPKEDELLLEQLDR 58
Ce-FLP-18 46 -----EKQDGRVFSKRDFDGAMPGVLRFGKRGGVWEKRESSVQKKEMPGVLRFGKRAY 98
L596_015629 59 PTWYDPEAYDTVKRAVGDETSMPGVLRFGKRGQAYVPF-GRLDKKEMPGVLRFGKRG- 114
Ce-FLP-18 99 FDEKKSVPGVLRFGKRSYFDEKKSVPGVLRFGKRDVPMDKREIPGVLRFGKRDYMADS 156
L596_015629 115 --EKKAVPGVLRFGKR-------EIPGVLRFGKRD------EIPGVLRFG-------- 149
Ce-FLP-18 157 FDKRSEVPGVLRFGKRDVPGVLRFGKRSDLEEHYAGVLLKKSVPGVLRFGRK 208
L596_015629 150 --KKSEMPGVLRFGKRNVPGVLRFGRK------------------------- 174

L596_014347	1	MLTAQLILA-CLLAVFACALG**YSSYPASE**-**TEDSRAVPFVPLPYGYWQSPQQFLDTNDE**	57
Ce-FLP-19	1	-MSFQLTLFSMLFLLIAVVVGQPIQSQNGDLKMQAVQDNSPLNMEAFNDDSALYDYLEQ	58
L596_014347	58	**LDDSQRL**KR**SKWASQIRYG**KR**ASWASQVRFG**	88
Ce-FLP-19	59	SDPSLKSMEKRWANQVRFGKRASWASSVRFG	90

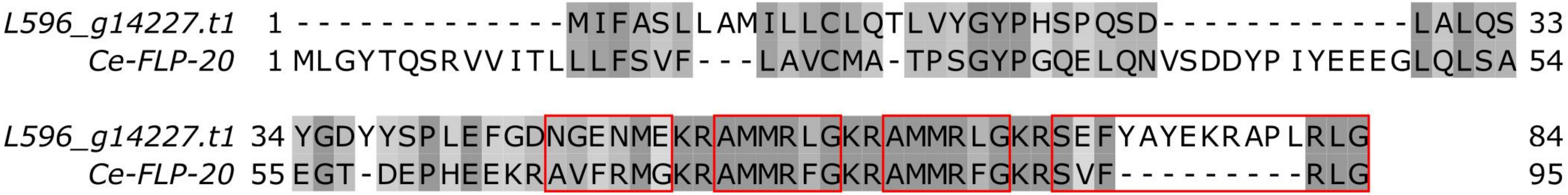
L596_g14227.t1 1 -------------MIFASLLAMILLCLQTLVYGYPHSPQSD------------LALQS 33
Ce-FLP-20 1 MLGYTQSRVVITLLLFSVF---LAVCMA-TPSGYPGQELQNVSDDYPIYEEEGLQLSA 54
L596_g14227.t1 34 YGDYYSPLEFGDNGENMEKRAMMRLGKRAMMRLGKRSEFYAYEKRAPLRLG 84
Ce-FLP-20 55 EGT-DEPHEEKRAVFRMGKRAMMRFGKRAMMRFGKRSVF----------RLG 95

```
L596_028079  1 MVAVVRLLLAAFLVLFSVFVAPARSAPTSVDDQQYRLLSRYLSQFNAPEYDPSGYMYFD 59
  Ce-FLP-21  1 ---------MRLFILLSCLLAWVLAAPYIDQEDALRVLNAYLEQFGPGSDRVYYVAEDD 50

L596_028079 60 QRSMKRGLGPRPLRFG 75
  Ce-FLP-21 51 HGSMKRGLGPRPLRFG 67
```

```
L596_029083  1 MNTSWTVAVFALFFACLLACSDAAFFDVFSPQLAPMDNM------DRAVRAPNVKWMRF 53
  Ce-FLP-22  1 MNRS----MIALCVVLMVSLVSAQVFDLDGQQLAGLEQNDARLMEQQVKRSPSAKWMRF 55

L596_029083 54 GKRAPAAKWMRFGKRAPAAKWMRFGKRSDSQENFGFPAEAEYNTL 98
  Ce-FLP-22 56 GKRSPSAKWMRFGKRSPSAKWMRFGKRSGAEAVS----EQDY--- 94
```

```
L596_024451   1 MVG-FSQLAILFFAVVVA-LIASNCEARPYDDEFGPSFYRPQYDAMRGFMGSRYLEKRV 57
 Ce-FLP-24    1 MLSSRTSSIILILAILVAIMAVAQCRNIQYDVEEMTPEA-----AFRYAQWGEIPHKRV 54

L596_024451  58 PNAADMMIRFGKRSGF                                            73
 Ce-FLP-24   55 PSAGDMMVRFGKRSI-                                            70
```

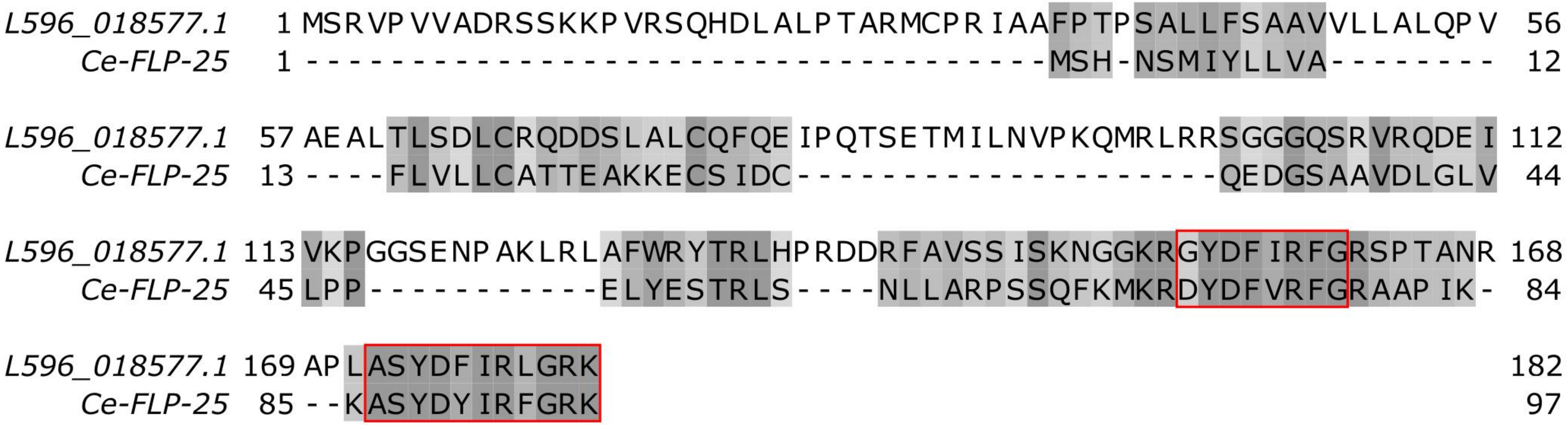

L596_018577.1 1 MSRVPVVADRSSKKPVRSQHDLALPTARMCPRIAAFPTPSALLFSAAVVLLALQPV 56
Ce-FLP-25 1 -----------------------------------MSH-NSMIYLLVA-------- 12
L596_018577.1 57 AEALTLSDLCRQDDSLALCQFQEIPQTSETMILNVPKQMRLRRSGGGQSRVRQDEI 112
Ce-FLP-25 13 ----FLVLLCATTEAKKECSIDC--------------------QEDGSAAVDLGLV 44
L596_018577.1 113 VKPGGSENPAKLRLAFWRYTRLHPRDDRFAVSSISKNGGKRGYDFIRFGRSPTANR 168
Ce-FLP-25 45 LPP-----------ELYESTRLS----NLLARPSSQFKMKRDYDFVRFGRAAPIK- 84
L596_018577.1 169 APLASYDFIRLGRK 182
Ce-FLP-25 85 --KASYDYIRFGRK 97

```
L596_027293  1 ---MKQLILWLVASFSILAL-LSRPVAALGSHQMARRDPGEFDSLLSELKSKAMGGRMR 55
  Ce-FLP-27  1 MFSLTQILTFLLVAITLMTFSSAQPIDEERPIFMERREASAFGDIIGELKGKGLGGRMR 59

L596_027293 56 FGKRASKASDFN-MDPDVYSPDRYLWLQ--                              82
  Ce-FLP-27 60 FGKRSSSPDISLAEMRAIYGGDQSNIFNFK                              90
```

```
L596_016308   1 -MSPRTLLLAILVLLISSSISSAAPNRILMRFGRSDPNLRPQN------AGLPSSFFRSF 53
 Ce-FLP-28    1 MFSVRSIFAIFCVLILALSTINAAPNRVLMRFGKRGGNSEGHLGYRFVPAGAPAI----- 55

L596_016308  54 DASRFGDDAPSAGGNYLYDPVE 75
 Ce-FLP-28   56 --AEYIDVDDVIGGDDRF---- 71
```

```
L596_944232  1 --------MRNLFCRLLLVSALIALIGSVSVRLRADKKAMRNSLVRFGKRGV----DDF 47
  Ce-FLP-32  1 MLSFVQTLILALLCSIVFVEA--------MPSMRPAKKAMRNSLVRFGKRADPVGTDDV 51

L596_944232 48 -MFPFNELSDPYEYKNPTPFPVAYVNRFSGSQDQF 81
  Ce-FLP-32 52 FLGESYGSADPYEYVPERM-----SNRGPSSVLLY 82
```

L596_009024 1 MQVALFLLVLLVVTVSLSE-AMFKHPIGLMAYSAAGGPKPLLRGYR-------PLLPDE 51
Ce-FLP-33 1 MR---FL-ILIVAIVLLSAVHGFSVEPRLAAFA-DGGAAELAQEARQARNAELEFIKRF 54

L596_009024 52 VEIKERR**APWGEGSEWEGMLQTLDNLRKPRFG**K 84
Ce-FLP-33 55 LPAKERRAPLEGFEDMSGFLRTIDGIQKPRFG- 87

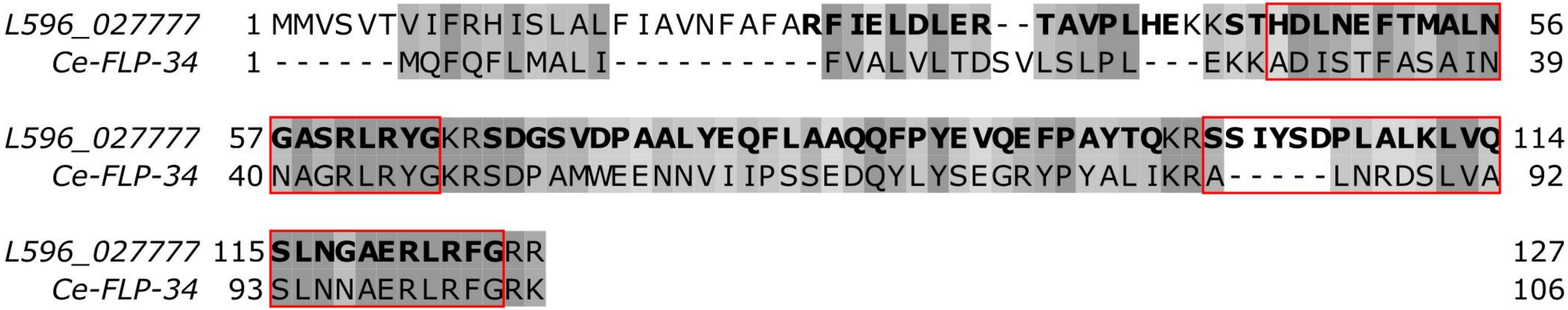

L596_027777 1 MMVSVTVIFRHISLALFIAVNFAFARFIELDLER--TAVPLHEKKSTHDLNEFTMALN 56
Ce-FLP-34 1 ------MQFQFLMALI----------FVALVLTDSVLSLPL---EKKADISTFASAIN 39
L596_027777 57 GASRLRYGKRSDGSVDPAALYEQFLAAQQFPYEVQEFPAYTQKRSSIYSDPLALKLVQ 114
Ce-FLP-34 40 NAGRLRYGKRSDPAMWEENNVIIPSSEDQYLYSEGRYPYALIKRA-----LNRDSLVA 92
L596_027777 115 SLNGAERLRFGRR 127
Ce-FLP-34 93 SLNNAERLRFGRK 106

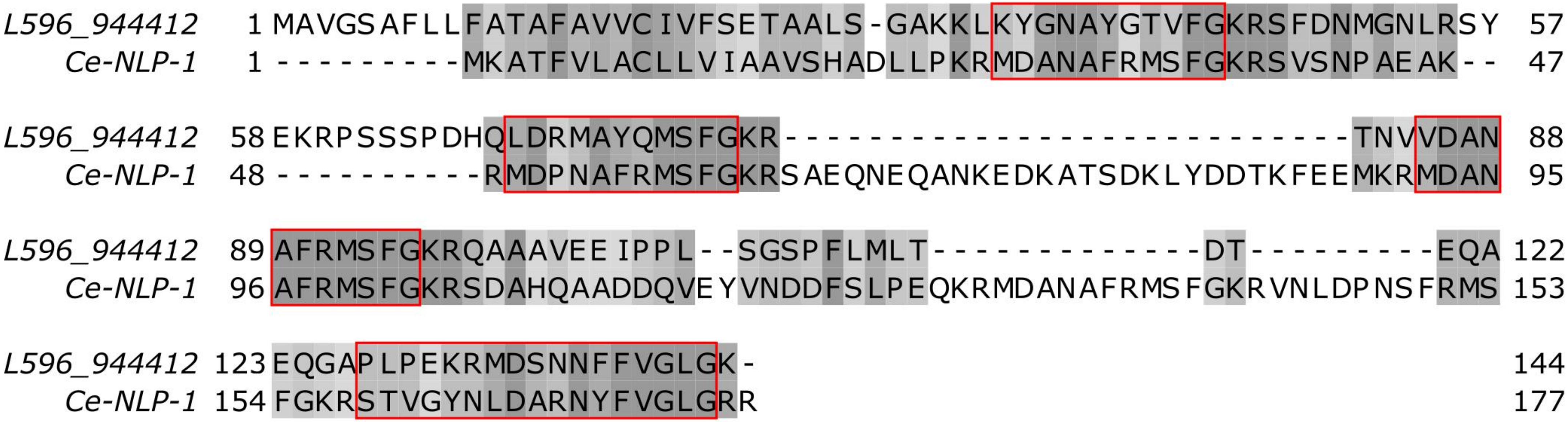
L596_944412 1 MAVGSAFLLFATAFAVVCIVFSETAALS-GAKKLKYGNAYGTVFGKRSFDNMGNLRSY 57
Ce-NLP-1 1 ----------MKATFVLACLLVIAAVSHADLLPKRMDANAFRMSFGKRSVSNPAEAK-- 47
L596_944412 58 EKRPSSSPDHQLDRMAYQMSFGKR------------------------------TNVVDAN 88
Ce-NLP-1 48 -----------RMDPNAFRMSFGKRSAEQNEQANKEDKATSDKLYDDTKFEEMKRMDAN 95
L596_944412 89 AFRMSFGKRQAAAVEEIPPL--SGSPFLMLT-------------DT----------EQA 122
Ce-NLP-1 96 AFRMSFGKRSDAHQAADDQVEYVNDDFSLPEQKRMDANAFRMSFGKRVNLDPNSFRMS 153
L596_944412 123 EQGAPLPEKRMDSNNFFVGLGK- 144
Ce-NLP-1 154 FGKRSTVGYNLDARNYFVGLGRR 177

```
L596_021176    1 ------MRAVSCFAFALAAF-----------LGTVVV----------------G----  21
L596_945649.2  1 -------------------------------------------MTRSVLRFWLVLSFF  15
Ce-NLP-2       1 MRATLVLFALLCAVYSEAVPLQVYRPDESSAVDVVVLENSPELYDSEDEDEWKQ----  54

L596_021176   22 AEQELVGVAMKRSLALGRMGFRPGKRSMEELLDE----EVEKRSLALGRMGFRPGKRS  75
L596_945649.2 16 ALAFLLVTAEKRSIALGRLSLRPGKRADYSSTAQDISDVLQFIR--------------  59
Ce-NLP-2      55 EEEFTEGAMGKRSIALGRSGFRPGKRSMDNFHTVDVSDLIMKRSMAMGRLGLRPGKR- 111

L596_021176   76 VVYDFVDPNELDGILDIITEDQMGKRSIALGRAGFRPAKRSLALGRTGFRPGKRSIAL 133
L596_945649.2    ----------------------------------------------------------
Ce-NLP-2     112 --------------------------SMAYGRQGFRPGKRSMAYGRQGFRPGKRSMAY 143

L596_021176  134 GRERFRPGKRSVNNMTPTTTVTPPFNVASGRCQLEKLQEVYQVLLKLAQGYEEMTKNC 191
L596_945649.2 60 ---------------------VPSVAPFCSGALVEAVTTNLEAVIRLLDKYSTYLERC  96
Ce-NLP-2     144 GRQGFRPGKRSNDMKEVFPQHVPEIYII------------------------------ 172

L596_021176  192 TAENPFQMPQ                                                 201
L596_945649.2 97 NE-LGYDIPI                                                 105
Ce-NLP-2         ----------
```

```
L596_944937  1 MNSITITVLALCVVGMV-VGKSLWADDDTVLIPLSESKGKRAINPFMDSIGKRSELPLI 58
   Ce-NLP-3  1 MSKIVACLVLLALSVMCVYSAPYEFRAKRAINPFLDSMGKRAVNPFLDSIGKRSFRPDM 59

L596_944937 59 YRFRPNHKRYFDSLAGQSLGKRTTMVVPYMAED-                          91
   Ce-NLP-3 60 ---ITEEKRYFDSLAGQSLGKRSNNRYEMLENYY                          91
```

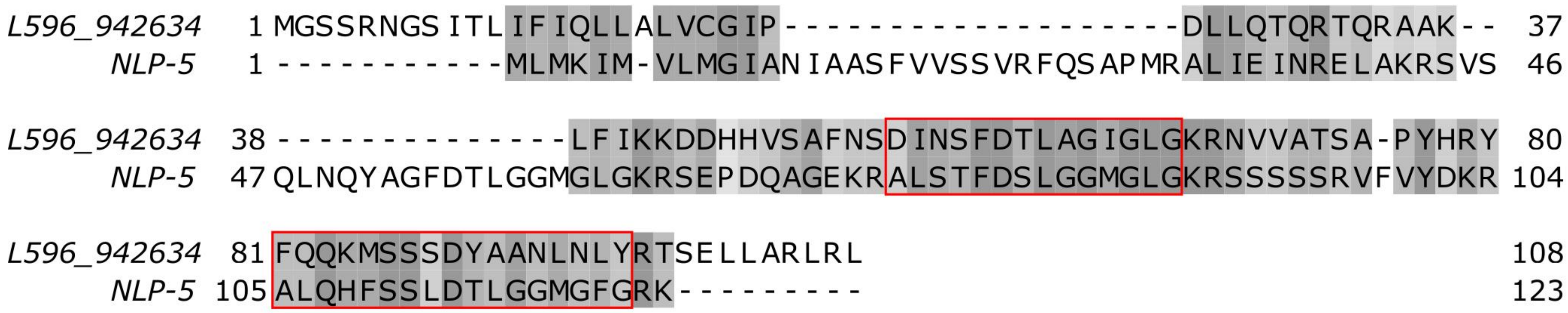
L596_942634 1 MGSSRNGSITLIFIQLLALVCGIP-------------------DLLQTQRTQRAAK-- 37
NLP-5 1 -----------MLMKIM-VLMGIANIAASFVVSSVRFQSAPMRALIEINRELAKRSVS 46
L596_942634 38 --------------LFIKKDDHHVSAFNSDINSFDTLAGIGLGKRNVVATSA-PYHRY 80
NLP-5 47 QLNQYAGFDTLGGMGLGKRSEPDQAGEKRALSTFDSLGGMGLGKRSSSSSRVFVYDKR 104
L596_942634 81 FQQKMSSSDYAANLNLYRTSELLARLRL 108
NLP-5 105 ALQHFSSLDTLGGMGFGRK--------- 123

```
L596_g8812.t1    1 MQFPPIRSFLIANSLDTIYSHLDTAHRCPQRFSYVCLATESSSKMGATQQVITVALT  57
L596_019467      1 --------------------------------------------------MFTLTAP   7
NLP-7b           1 -------------------------------------------------MYIKAALL   8

L596_g8812.t1   58 AFVFLALVDRFTSATYVYRTLTYPEDD--SGTQQRMEHQGQPMRVPLAYLAAQQ--- 109
L596_019467      8 AIVFLAVASRFTCDAYGLWSFYTNQRD--DAPV-KVGEGFDANNFPIPFEGR-----  56
NLP-7b           9 IVVLFGVASQITSALYLKQADFDDPRMFTSSFGKRSAIESEPQAYPKSYRAIRIQRR  65

L596_g8812.t1  110 -MNKNTD-----------YRLKRFSHPMLYENEKRSDFDFDDPRFLSSAFGKRRDPT 154
L596_019467     57 ------K-----------Y-SKRF--KPSYVLMKRDEFDFDDPRFFSTSFG------  87
NLP-7b          66 SMDDLDDPRLMTMSFGKRMILPSLADLHRYTMYDKRGSDIDDPRFFSGAFGRK---- 118

L596_g8812.t1  155 W                                                         155
L596_019467        -
NLP-7b             -
```

```
L596_008109   1 MRTNNLGHVPLTLLSLT---VTLAYSLPYAVVVGSNTAPSIVPSGHLTSKRAFDRLDM  55
   Ce-NLP-8   1 MSQKLLPISPLQLLFLQCLLIGFTAAYPYLIFPAS---PSS-GDSRRLVKRAFDRFDN  54

L596_008109  56 S-PFDFGAYRKRAFDRLDESAFGFTRKRRAFDRLDESVFGLMAARRRRAFDRLEQSG- 111
   Ce-NLP-8  55 SGVFSFGAKRFDRYDD--ETAYGYGFDNHIFKRS-ADPYRFMSVPTKKAFDRMDNSDF 109

L596_008109 112 FGLVKKRSFDRLDSGNFGFGMGKRSGNYVIPAHALAKRPFDRLERSPFGLSKRSQKIA 169
   Ce-NLP-8 110 FGAKRKRSFDRMGGTEFGLMKRSAPESREQL--------INNLAESIITLR-RAREAE 158

L596_008109 170 LGPEVVDLLGEFRPSAVDF 188
   Ce-NLP-8 159 SSPESQRTIITYDD----- 172
```

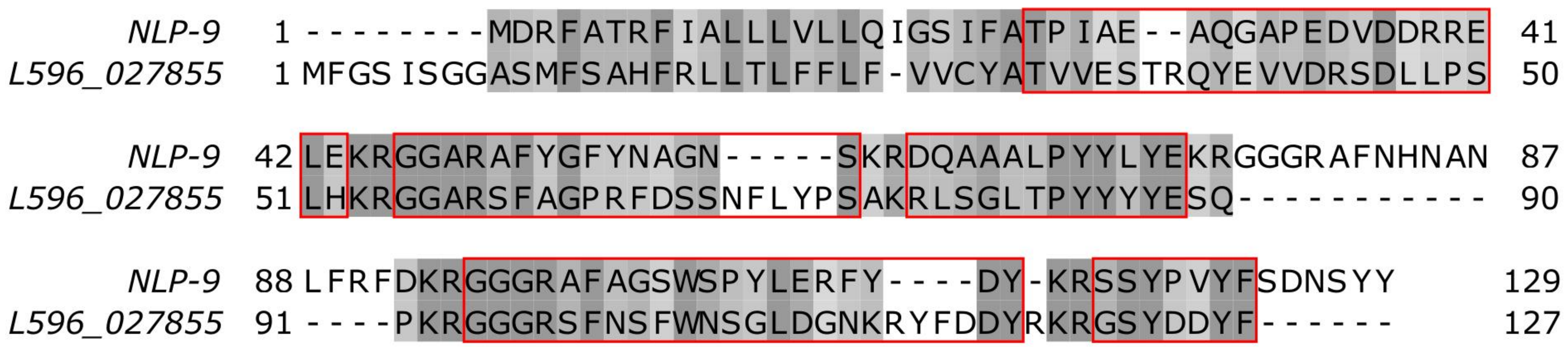
NLP-9 1 --------MDRFATRFIALLLVLLQIGSIFATPIAE--AQGAPEDVDDRRE 41
L596_027855 1 MFGSISGGASMFSAHFRLLTLFFLF-VVCYATVVESTRQYEVVDRSDLLPS 50
NLP-9 42 LEKRGGARAFYGFYNAGN-----SKRDQAAALPYYLYEKRGGGRAFNHNAN 87
L596_027855 51 LHKRGGARSFAGPRFDSSNFLYPSAKRLSGLTPYYYYESQ----------- 90
NLP-9 88 LFRFDKRGGGRAFAGSWSPYLERFY----DY-KRSSYPVYFSDNSYY 129
L596_027855 91 ----PKRGGGRSFNSFWNSGLDGNKRYFDDYRKRGSYDDYF------ 127

```
L596_030545   1 MSVATLNLLFVVALQLALFVRSEEVHNMLQAYADNPKVLV---PSELFAADKR--ASL  53
  Ce-NLP-10   1 --------MWYIALLLAVIATSVT----AQKADDEPIVFLVRVPIDEMDDDSSLLESY  46

L596_030545  54 PYSGGFYGKRAVPFSGGLYGKRAPLPYSGGFYGKRAALPYSGGLYGKRTMPFNGGFYG 111
  Ce-NLP-10  47 YHPRDILSKRAIPFNGGMYGKRSTMPFSGGMYGKRSGQIF----------------AQ  88

L596_030545 112 KRAALPYSGGLYGKRTQVPFSGG----LYGKRAMPLNGGFYGKRSENRIAVRALPISG 165
  Ce-NLP-10  89 RRAAIPFSGGMYGKRSLVPQSYSNNENQIKRGAMPFSGGMYGR--------------- 132

L596_030545 166 GFFG                                                       169
  Ce-NLP-10     ----
```

```
L596_010921  1 MLLTGSSWVYSMLMLIVVTFLLLSMGQADSEPSRFNRQDRDYRPLQFGKREGFRPLQFG 59
  Ce-NLP-12  1 -MLRHHSCAL--LMLI-----LVFVEVFATQSPTFDRQDRDYRPLQFGKRDGYRPLQFG 51

L596_010921 60 KRGDYRPLQFGKRASDAMPTLYAYPEYL--                               87
  Ce-NLP-12 52 KR-DYRPLQFGKRSSGSSGPVVLEPIWEWQ                               81
```

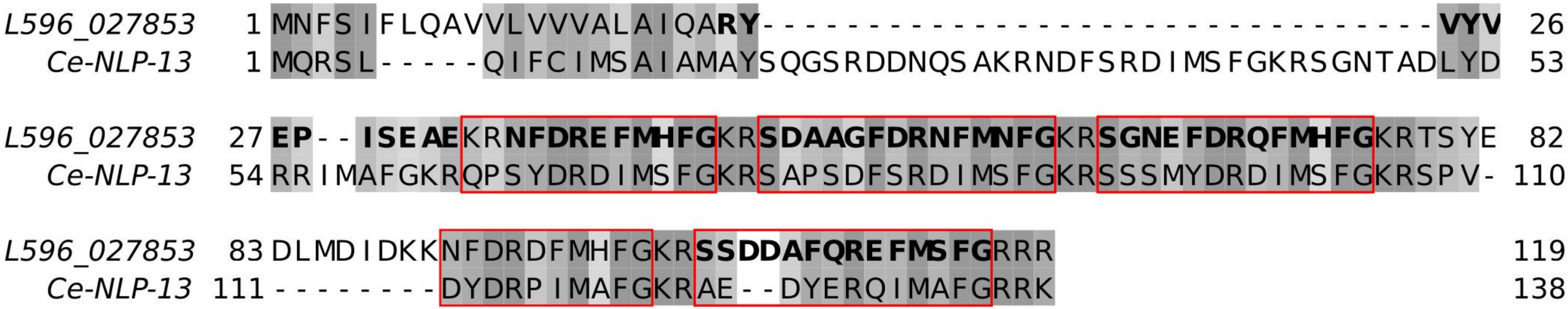
L596_027853 1 MNFSIFLQAVVLVVVALAIQARY--------------------------------VYV 26
Ce-NLP-13 1 MQRSL-----QIFCIMSAIAMAYSQGSRDDNQSAKRNDFSRDIMSFGKRSGNTADLYD 53
L596_027853 27 EP--ISEAEKRNFDREFMHFGKRSDAAGFDRNFMNFGKRSGNEFDRQFMHFGKRTSYE 82
Ce-NLP-13 54 RRIMAFGKRQPSYDRDIMSFGKRSAPSDFSRDIMSFGKRSSSMYDRDIMSFGKRSPV- 110
L596_027853 83 DLMDIDKKNFDRDFMHFGKRSSDDAFQREFMSFGRRR 119
Ce-NLP-13 111 --------DYDRPIMAFGKRAE--DYERQIMAFGRRK 138

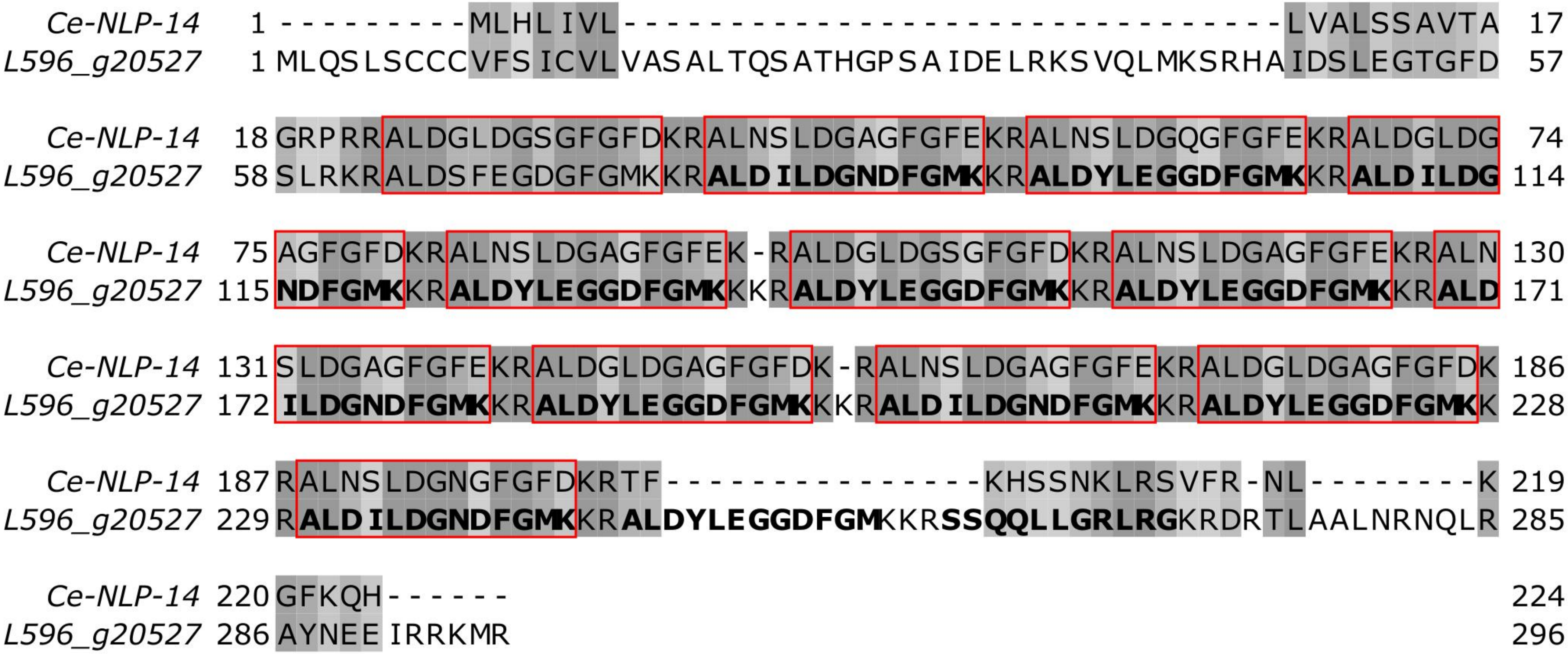
Ce-NLP-14 1 ----------MLHLIVL-----------------------------LVALSSAVTA 17
L596_g20527 1 MLQSLSCCCVFSICVLVASALTQSATHGPSAIDELRKSVQLMKSRHAIDSLEGTGFD 57
Ce-NLP-14 18 GRPRRALDGLDGSGFGFDKRALNSLDGAGFGFEKRALNSLDGQGFGFEKRALDGLDG 74
L596_g20527 58 SLRKRALDSFEGDGFGMKKRALDILDGNDFGMKKRALDYLEGGDFGMKKRALDILDG 114
Ce-NLP-14 75 AGFGFDKRALNSLDGAGFGFEK-RALDGLDGSGFGFDKRALNSLDGAGFGFEKRALN 130
L596_g20527 115 NDFGMKKRALDYLEGGDFGMKKKRALDYLEGGDFGMKKRALDYLEGGDFGMKKRALD 171
Ce-NLP-14 131 SLDGAGFGFEKRALDGLDGAGFGFDK-RALNSLDGAGFGFEKRALDGLDGAGFGFDK 186
L596_g20527 172 ILDGNDFGMKKRALDYLEGGDFGMKKKRALDILDGNDFGMKKRALDYLEGGDFGMKK 228
Ce-NLP-14 187 RALNSLDGNGFGFDKRTF---------------KHSSNKLRSVFR-NL--------K 219
L596_g20527 229 RALDILDGNDFGMKKRALDYLEGGDFGMKKRSSQQLLGRLRGKRDRTLAALNRNQLR 285
Ce-NLP-14 220 GFKQH------ 224
L596_g20527 286 AYNEEIRRKMR 296

```
L596_009544    1 MVASSVLYSSLGAVCVLAVVSIFAVEST--QAAP----TLSHRPHSLSQVKRAFDSLT  52
L596_009545    1 ---------------MRHKRFYAGLEVT--QAAP----TLSHRPHSLSQVKRAFDSLT  37
 Ce-NLP-15     1 MPSSSS-SSSFFAAVLLVIVMMSTVESAAVRLRPVGSLFFLNRP----HEKRAFDSLA  53

L596_009544   53 GSGFS-GFDKRAFDSFMGSGFTGMDKRSFDSLVGSGFTGMDKRGFDALTSSGFTGFDK 109
L596_009545   38 GSGFS-EFDKLAFDSFMGFGFTGMDKRGFHDLARIVLKALTL----------------  78
 Ce-NLP-15    54 GSGFDNGFNKRAFDSLAGSGFGAFNKRAFDSLAGSGFGAFNKRAFDSLAGSGFSGFDK 111

L596_009544  110 RSFDSLNGVGFTGFD-----------------                           124
L596_009545      --------------------------------
 Ce-NLP-15   112 RAFDSLAGQGFTGFEKRAFDTVSTSGFDDFKL                           143
```

```
L596_028624  1 MLSLTVLAKFLALAIVLCSVAISALPYSDG--AESPYMEPYLNQFESSLSYQPRVRKAGH 58
 Ce-NLP-17   1 ---------MFSKSILFCLLVLVFNVFGANFENDQDVMRP-----------PFQALKRGS 40

L596_028624 59 LSNMMRIGRSDPWMNRETRESADNNAN----SLRGKIWLLP--                  95
 Ce-NLP-17  41 LSNMMRIGKRQMSRQQEYVQFPNEGVVPCESCNLGTLMRIGRR                  83
```

```
L596_943837    1 MQSTLCA-LALCSFAVVLLAGEITELGSDSSAAS----VDKR-GLHTFAFAKRYPMSG  52
  Ce-NLP-18    1 MNANVYSIVYFLSFLVLCISA---QLHADSGATEVDGIVDKRSPYRAFAF---------  47

L596_943837   53 FAKRDDPELPAEPSEKELFGDDLEEQKRGWNRFAFAKRS-MRNFAFAKRAVRPFAFAK 109
  Ce-NLP-18   48 -AKRSDEENLD-----------F---LEKRARYGFAKRSPYRTFAFAKR---------  81

L596_943837  110 KSMRNFAFAKRGAYSSFA                                          127
  Ce-NLP-18   82 ASPYGFAFAKRGQFSSFA                                          100
```

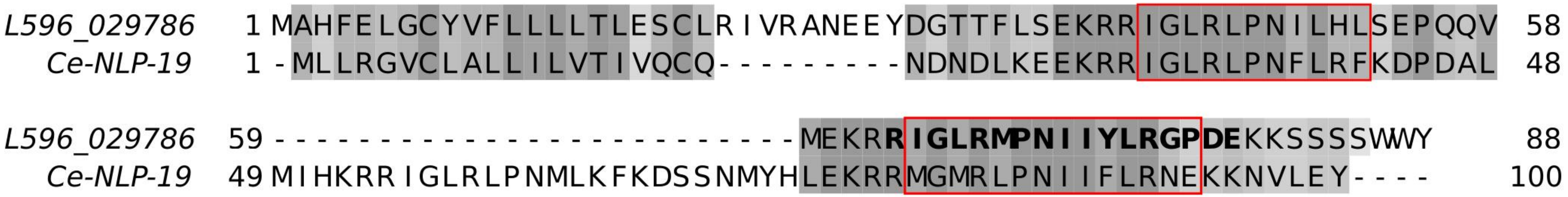

L596_029786 1 MAHFELGCYVFLLLLTLESCLRIVRANEEYDGTTFLSEKRRIGLRLPNILHLSEPQQV 58
Ce-NLP-19 1 -MLLRGVCLALLILVTIVQCQ---------NDNDLKEEKRRIGLRLPNFLRFKDPDAL 48
L596_029786 59 -------------------------MEKRRIGLRMPNIIYLRGPDEKKSSSSWWY 88
Ce-NLP-19 49 MIHKRRIGLRLPNMLKFKDSSNMYHLEKRRMGMRLPNIIFLRNEKKNVLEY---- 100

```
L596_018926    1 MPETVRFSRSSLLLATLLFGVAAS----------SPGEVEKRGGGR----------SF  38
  Ce-NLP-21    1 --------MRNSLFTTLFFGLAALVMVLNAQYTSELEEDEKRGGARAMLHKRGGARAF  50

L596_018926   39 QGFSGDK--RGGGHLFAGSY---TMQPNDEEKRGGARVFMPVSEDKRGGARVFVPVGQ  91
  Ce-NLP-21   51 SADVGDDYKRGGARAFYDEKRGGARAFLTEMKRGGARVFQGFEDEKRGGARAFM---- 104

L596_018926   92 EEKRGGGHVFVPLMAQEKRGGGRLFQLNDKRAGGRGFQMLPSKKLFSEWYLLEDPTEV 149
  Ce-NLP-21  105 ---------------MDKRGGGRAFGDMMKRGGARAFV--ENSKRDEDWVIRPFE--- 142

L596_018926  150 LMPAEEKRAGGRTFPVHGDDSKEKRVWEELESYFP                        184
  Ce-NLP-21  143 -DDRLEKRGGGRSFPVKPGRLDD------------                        165
```

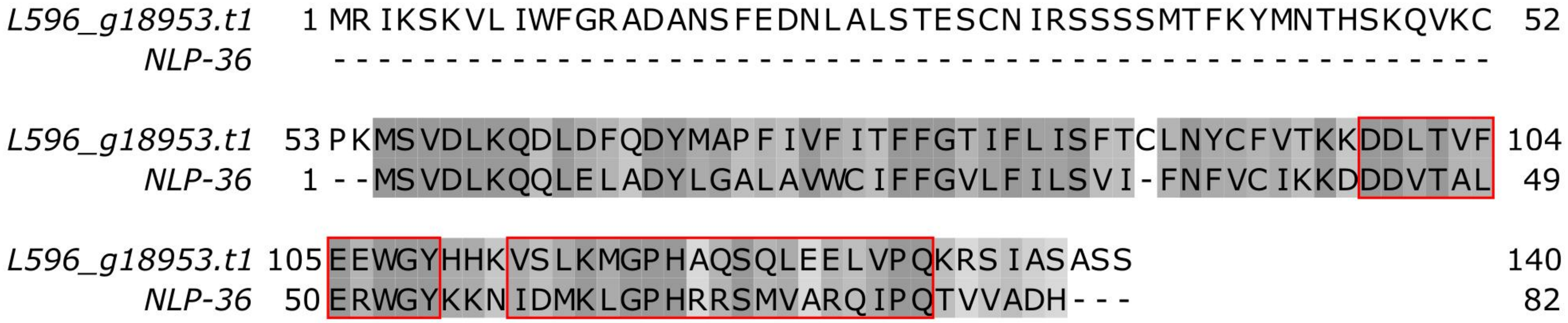
L596_g18953.t1 1 MRIKSKVLIWFGRADANSFEDNLALSTESCNIRSSSSMTFKYMNTHSKQVKC 52
NLP-36 --
L596_g18953.t1 53 PKMSVDLKQDLDFQDYMAPFIVFITFFGTIFLISFTCLNYCFVTKKDDLTVF 104
NLP-36 1 --MSVDLKQQLELADYLGALAVWCIFFGVLFILSVI-FNFVCIKKDDDVTAL 49
L596_g18953.t1 105 EEWGYHHKVSLKMGPHAQSQLEELVPQKRSIASASS 140
NLP-36 50 ERWGYKKNIDMKLGPHRRSMVARQIPQTVVADH--- 82

L596_g9328.t: 1 MKV-------LFCFLLALFAVVSIALPMAKSQHGQLLRQLLDRFPVDEFHPLERRFASL 52

Ce-NLP-37 1 MSSRISVSLLLLAVVATMFFTANVV-DATPRSQGNMMR-YGNSLPAYAPHVL-YRFYNS 56

L596_g9328.t: 53 IEDRVMNKR**NNAEVVNGILKNFGALDRLGDVG**K 85

Ce-NLP-37 57 RQFAPINKRNNAEVVNHILKNFGALDRLGDVGK 89

```
L596_007721   1 MHPSLVDSFRVFVLLLTLVVAIASSAAVPSFEDSPMSIGTFVKRSNWNKANGLWGKRA  58
 Ce-NLP-38    1 ------------MQLIHFIVGLAMLISLS-------LAASDDRVLGWNKAHGLWGKRS  39

L596_007721  59 QADPQEMTMWKRPEDWTKLNSLWGKRSSWSTANGLW----------------------  94
 Ce-NLP-38   40 VQEASQ--DKRTPQNWNKLNSLWGKRSASSFDDDYTTENGDDDVTMLYKRSNLSPRFL  95

L596_007721  95 ----------GKRASWQTANGLWGKRSSPPMNLDDTVY 122
 Ce-NLP-38   96 GRMTFARIPKISPAQWQRANGLWGR------------- 121
```

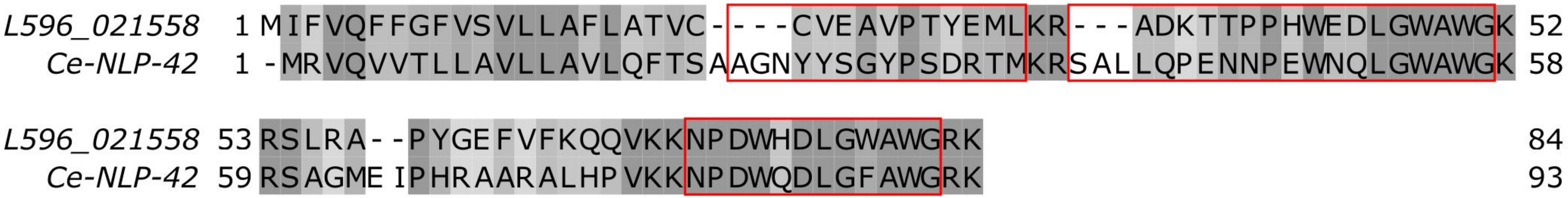

L596_021558 1 MIFVQFFGFVSVLLAFLATVC----CVEAVPTYEMLKR---ADKTTPPHWEDLGWAWGK 52
Ce-NLP-42 1 -MRVQVVTLLAVLLAVLQFTSAAGNYYSGYPSDRTMKRSALLQPENNPEWNQLGWAWGK 58
L596_021558 53 RSLRA--PYGEFVFKQQVKKNPDWHDLGWAWGRK 84
Ce-NLP-42 59 RSAGME IPHRAARALHPVKKNPDWQDLGFAWGRK 93

L596_018923 1 --MESGRFFATVIFVFCVLLAHIIC---CSEA**SMQPVEARPAQI**KRFYSWEEGKR**SSG** 53
Ce-NLP-43 1 MSLAQSTFYL----LFVAFLAVVIAVTADKQQSAYDLETPISAYKRFYSWEDAKRAAS 54

L596_018923 54 **DAFADFDSLPLERMF**KRRFYAWASHFDKPHFDT---P----------QQQQF------ 92
Ce-NLP-43 55 SEEG-------MRNKRKQFYAWAGKRSSAPVHYFEDSIAQEAGPSMEKRKQFYAWAGK 105

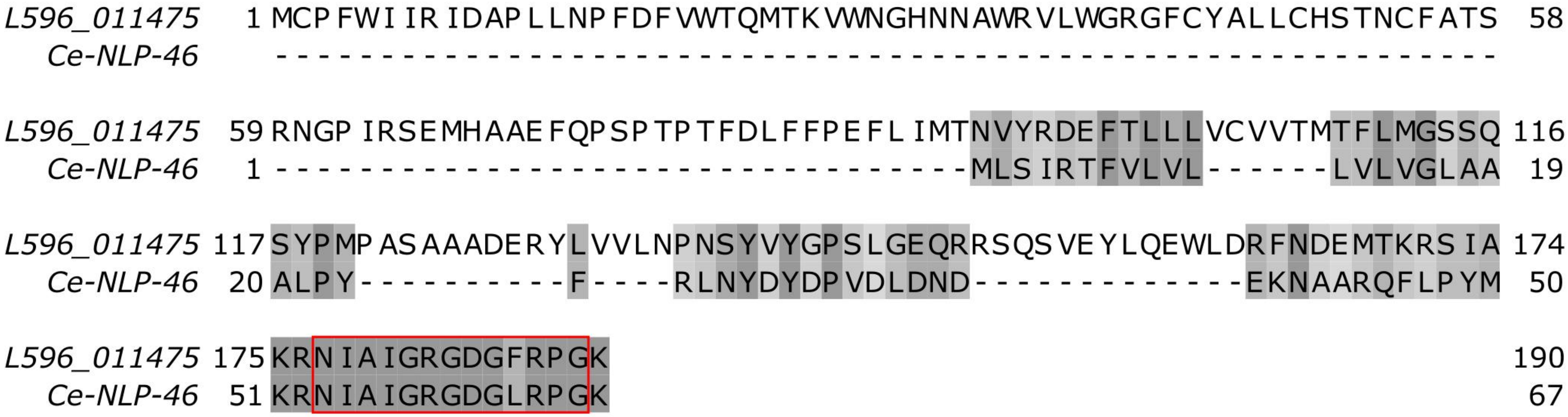

L596_011475 1 MCPFWIIRIDAPLLNPFDFVWTQMTKVWNGHNNAWRVLWGRGFCYALLCHSTNCFATS 58
Ce-NLP-46 --
L596_011475 59 RNGPIRSEMHAAEFQPSPTPTFDLFFPEFLIMTNVYRDEFTLLLVCVVTMTFLMGSSQ 116
Ce-NLP-46 1 ---------------------------------MLSIRTFVLVL------LVLVGLAA 19
L596_011475 117 SYPMPASAAADERYLVVLNPNSYVYGPSLGEQRRSQSVEYLQEWLDRFNDEMTKRSIA 174
Ce-NLP-46 20 ALPY----------F----RLNYDYDPVDLDND-------------EKNAARQFLPYM 50
L596_011475 175 KRNIAIGRGDGFRPGK 190
Ce-NLP-46 51 KRNIAIGRGDGLRPGK 67

```
L596_945015  1 -MKNCFFAVFCFFVLISQIMSWPAGESADALPDELLARVYRTVLLKAKRSPSIGLSLAE 58
 Ce-NLP-49   1 MMKWLLLAVFCIA-----AYAWADGTE-DSNIDQLMSRVYRTVLLKSKRSPSMGLSLAE 53

L596_945015 59 YMASPQGQDNFHFIPSGRK 77
 Ce-NLP-49  54 YMASPQGGDNFHFMPSGRK 73
```

```
L596_g10891.t.  1 MRLLGLLIAITLLLLVVGSDAFFQRFIQSRHRKNTGTPAGMEFESNVQPFIRFRKSFI 58
    Ce-NLP-51   1 MRFLIL----ALLVLFAITQAYPSSDYQPRYR------KSQTQEANIQPFIRFRKSTQ 48

L596_g10891.t. 59 YPS-----PNLR-AFDGYGSRENDQLEDLLFQARAAAAAASQK 95
    Ce-NLP-51  49 PQHNWMFRPDVAPYFE--------------------------- 64
```

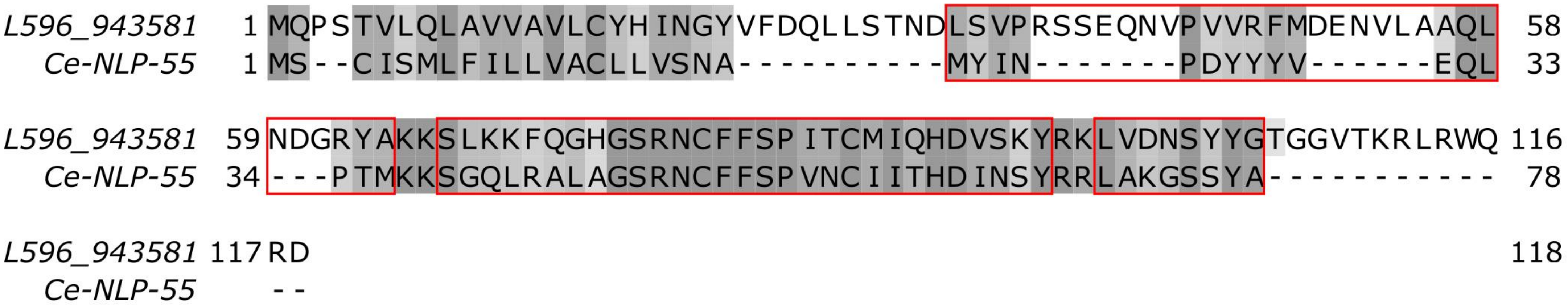
L596_943581 1 MQPSTVLQLAVVAVLCYHINGYVFDQLLSTNDLSVPRSSEQNVPVVRFMDENVLAAQL 58
Ce-NLP-55 1 MS--CISMLFILLVACLLVSNA----------MYIN-------PDYYYV------EQL 33
L596_943581 59 NDGRYAKKSLKKFQGHGSRNCFFSPITCMIQHDVSKYRKLVDNSYYGTGGVTKRLRWQ 116
Ce-NLP-55 34 ---PTMKKSGQLRALAGSRNCFFSPVNCIITHDINSYRRLAKGSSYA----------- 78
L596_943581 117 RD 118
Ce-NLP-55 --

```
L596_g10365.t1     ---------------------------------------------------------
L596_947118      1 MNPAVLTRLKNSLALLDKSVNAPPGLAKPFRHNRTVSDMKENVFPDARLRDVKCIIL  57
Ce-NLP-56c         ---------------------------------------------------------

L596_g10365.t1   1 ---------------MGSTLMGAHSAFLLIVI-------YSVILAAVCSEGKTAEAL  35
L596_947118     58 AEFICVFNSYFVCVENGFHSHGCPFGVPSHFI-------YSVILAAVCSEGKTAEAL 107
Ce-NLP-56c       1 --------------------MPSPSSLLGSLLLVCAVLTITSRASSIMTDDVEPPQL  37

L596_g10365.t1  36 LDRQARSFPYSMPFIGMLRPQSGPGVYVPIRSPLAENYQMARELAELVDSLDEQQNF  92
L596_947118    108 LDRQARSFPYSMPFIGMLRPQSGPGVYVPIRSPLAENYQMARELAELVDSLRRTAEL 164
Ce-NLP-56c      38 LTRQLRSFPYSVSFYRMLGHDRQLRP----------YYGVNDEVAALIDSMNSDNVA  84

L596_g10365.t1  93 R----RQRRDDTVVYKRYACRFK-----------------FCRIFDA---------- 118
L596_947118    165 Q----TSAQGRHCGLQEVCVPIQVLSHLRRVSYSLKSPNVVFFFITNAKQDQQEIQS 217
Ce-NLP-56c      85 NEDVFPTRPRRSDGLRGSFYWAR---------------FVVCQPVLVI--------- 117

L596_g10365.t1     -----------------
L596_947118    218 QGTPKM-SVQTTVAIRL                                         233
Ce-NLP-56c         -----------------
```

```
L596_015717  1 MQLFSFVASLFVVLFALLPNFSALPMIASPNSKAHNYISSYDFEPNSDEAAFLAAFQQFR 60
  Ce-NLP-61  1 --MSHWIATLLAF-ILIANAFAALST-------------GADVGASDDV---RSYVMPFY 41

L596_015717 61 PMQKKWNRLEPSIRFF------------------                           76
  Ce-NLP-61 42 GMEKKWSRREPSIRFFKRNGGGQDLPPSRFLWDY                           75
```

```
L596_945860  1 MCGLASLLLL-----LALTSALPLGEAFFPSAGFYVPVSYERFPDGVVKGQVKRNNAL  53
 Ce-NLP-64   1 MMAQKTLIIAVMLVCSILQPMLALG-SLTPSAAFRAN--------------MQQRERS  43

L596_945860 54 APEIVYVPAYGDGRTLFDVPKRDFPLQRQQLKRMKPCFYSPIQCLMKRAP 103
 Ce-NLP-64  44 PNTLFYM----DGAS----KQYGDEIKDPIYKRFKPCYYSPIQCLIKRK-  84
```

L596_019315.1 1 MQ--------------------------------------PVRALILLLVAFVGFLAA 20
Ce-NLP-66 1 MSSGKLFQFFIVFLATLLLADAIPMVSSRDEDDQIIQKRLSNDALIRLLMRNRGTQTQ 58

L596_019315.1 21 **FPAERQWQNAKFP**RRAALLMKRREEQALRNCFFSPVQCLLPINDKALRKFVPHN 74
Ce-NLP-66 59 LGLKR------GLVKKAEVERRSIDEDFSNCFLSPVQCMLPSSRK--------- 97

```
L596_944292   1 -----------------------------------------MKTVILVTLLFSIIWS  16
L596_946112   1 ----------------------------------------MFPNVRALSIMFMICIA  17
  Ce-NLP-67   1 -------------------------------------------MRVLTFLLVTLFAL  14

L596_944292  17 AAVISALPHQRD---LYDVLLNDLERTLEEQQLMDR-------------FERRARRA  57
L596_946112  18 SQFSTALPQNRILRSLYSANSVDTSGNMESY-----------LVPGYASGMDKRVPL  63
  Ce-NLP-67  15 ANVMQAQRYDRA---IYEALLNDLEREFVERELAQHVLEKRELLRQDRQELDRVRRA  68

L596_944292  58 SEKKSYPRMCYFSPIQCLFTRN-----------------                   79
L596_946112  64 SLGKKYDRNCFFSPVQCMLSFNNPANQHDDAIWNSRGRR                  102
  Ce-NLP-67  69 SEKKSYPRNCYFSPIQCLFTRN-----------------                   90
```

```
L596_021905   1 ---MPGVNLFVLILALIVASVGVAQSLPAFAVDTLNNSDLKAFIGDPVWNLRLKSL-I  54
 Ce-NLP-69    1 MHFFPILLLSILLILISTCSSTLVNNSPTAAFDTNAYSELNAKAKASM--RRLADILD  56

L596_021905  55 EEELQRRFAFEPDYAYFAD------------SFNNIDMPAKRYSRIGGTIVMG-   95
 Ce-NLP-69   57 FEMYQRRLSAAPDNSYYYQISPPHHQRISSLPLTIFPLPDKRLHRIGGNIVMGK  110
```

L596_011324 1 ----MTKDQQILAVLVASLCFVAAFAEPATPSRATR**AALGGADPFDQSRYYM**KVKKWY 54
Ce-NLP-70 1 MSVSRPLNLAILSILVGLLYLSACTC---------------APSVSAHHLGLRLKKWY 43

L596_011324 55 DWNDSNLQVDKKWYDWQSIPTNDKRHHNEKRQFGNVLRNSRFEWSRM 101
Ce-NLP-70 44 EWNN-DMEITKKWYDWQNVPHALQQKRQPFETSDYIIE--------- 81

L596_945877	1	MNVDQSTSFSSMVSAFIFALMALLLCNAVNA**TNVPVPLSAFIDKPLASEALPQAMYPW**	58
Ce-NLP-71		--	
L596_945877	59	**AQQRPYSAYG**KRSHQVELR**SMSQFKNCYFSPIQCVLME**RRRR	100
Ce-NLP-71	1	--MLRPNRYWKRAHNIDTRALNQFKNCYFSPIQCVLMERRRK	40

```
L596_018881  1 MPSSASLVRFVVVVLAVLLVLASASDNDTPDKRAAYLSRYGRAVLSRYGKRSDPQFEVD 59
  Ce-NLP-72  1 -----MLTRVPVLILAVIVMLALCQEPEKPEKRPALLSRYGRAVLPRYGKRSGNLMESS 54

L596_018881 60 NT--------VGGSRMDGLYFCRWFDGEVMRCRPYMSQ                      89
  Ce-NLP-72 55 QNSLTEESSDVVCQLIDGKYICLPVDA--VRFRPFFL-                      90
```

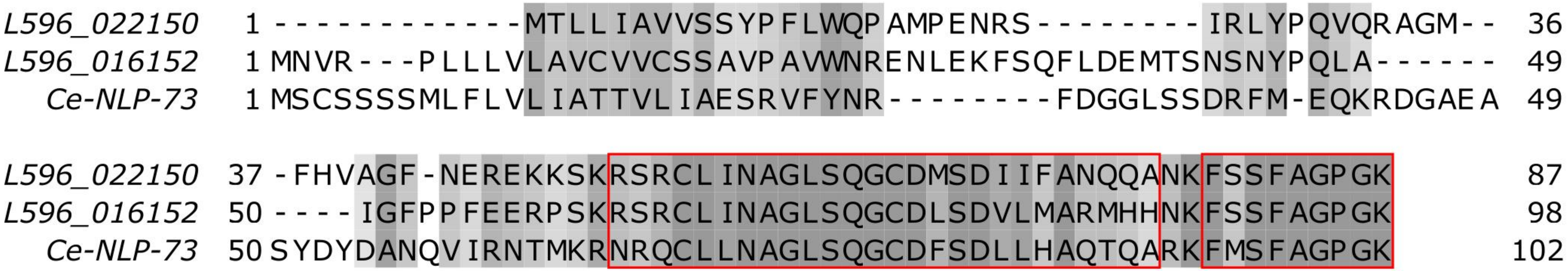
L596_022150 1 ------------MTLLIAVVSSYPFLWQPAMPENRS--------IRLYPQVQRAGM-- 36
L596_016152 1 MNVR---PLLLVLAVCVVCSSAVPAVWNRENLEKFSQFLDEMTSNSNYPQLA------ 49
Ce-NLP-73 1 MSCSSSSMLFLVLIATTVLIAESRVFYNR--------FDGGLSSDRFM-EQKRDGAEA 49
L596_022150 37 -FHVAGF-NEREKKSKRSRCLINAGLSQGCDMSDIIFANQQANKFSSFAGPGK 87
L596_016152 50 ----IGFPPFEERPSKRSRCLINAGLSQGCDLSDVLMARMHHNKFSSFAGPGK 98
Ce-NLP-73 50 SYDYDANQVIRNTMKRNRQCLLNAGLSQGCDFSDLLHAQTQARKFMSFAGPGK 102

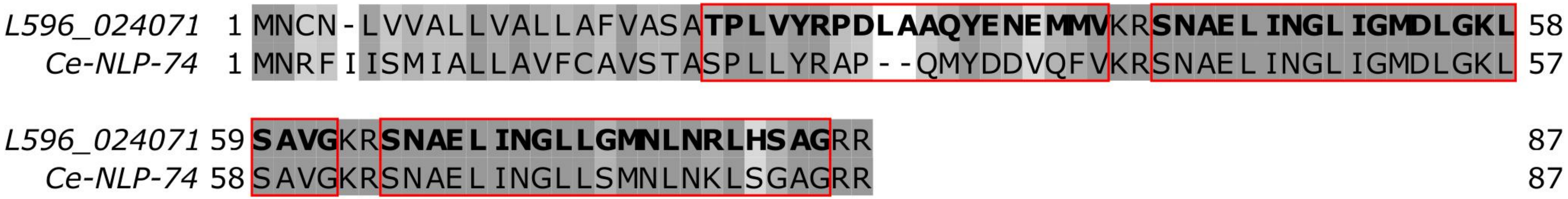
L596_024071 1 MNCN-LVVALLVALLAFVASATPLVYRPDLAAQYENEMMVKRSNAELINGLIGMDLGKL 58
Ce-NLP-74 1 MNRFIISMIALLAVFCAVSTASPLLYRAP--QMYDDVQFVKRSNAELINGLIGMDLGKL 57
L596_024071 59 SAVGKRSNAELINGLLGMNLNRLHSAGRR 87
Ce-NLP-74 58 SAVGKRSNAELINGLLSMNLNKLSGAGRR 87

```
L596_944742   1 MQ-LTCTFLCFVLIGASLGCFLSSCPYRRYGRNVRCSSCGADMNGVCVAESICCTSES  57
 Ce-NLP-75    1 MGSSPILLVLAISIGLASACFLNSCPYRRYGRTIRCSSCGIENEGVCISEGRCCTNEE  58

L596_944742  58 CSEDLSCTDAAVCPPRLCKLGGVAGFCIAPAMCCTQSRVTFVSRITIAFELLRPSRKV 115
 Ce-NLP-75   59 CFMSTECSYSAVCPELFCKIGHHPGYCMKKGYCCTQGGCQTSAMC------------- 104

L596_944742 116 TRSMYKRKQLFDLLHYPNCTIDSLFNINKINALQCVWVGQEDRVEDPEADRQEFESLE 173
 Ce-NLP-75      ----------------------------------------------------------

L596_944742 174 GVT                                                        176
 Ce-NLP-75      ---
```

```
L596_942816   1 MNKVLVVFA--AFAVLAVFGQ---EPPPPPFLQGAPPATIQSFNALIQGAGGKTDKEI  53
 Ce-NLP-77    1 MC-RLAVFALLAVAAVSVYGQPAGGQDVPPFLRNATPAQLQSFQQLIQANGHLTETAL  57

L596_942816  54 DAAVEKWVAGQSAQIKTSFVKFKNEIKTQQAQAEAAHKAALGKFSPAAKAADAKLSAV 111
 Ce-NLP-77   58 DGKVQAWVNQQGGKVAADWADFQKFIKGQQGQAEAAHQAAVSNFSPAAKKADADLTAI 115

L596_942816 112 PPMSAPRSSRPCKVKLCLLNPRTATFRIV-------                       140
 Ce-NLP-77  116 SNDSSLSVQAKGQKIQAYLNSLPANVKAELEKAQGQ                       151
```

L596_945266 1 MTTSLLLNLLCSLLVLMLLADSPMTVKA**APDSEFFDRHPK**WT-RLTPSGGSLVSGRGNF 58
Ce-NLP-81 1 MLK----SSIFSLLIVLLMV---CSFGNVSANDFFLRSAKWVSKMNPSGGALVSGRGGF 52

L596_945266 59 RPGFYSHSQIDPRIYLSEPSFAFKRSSLPD- 88
Ce-NLP-81 53 RPGFVSRDWRH---AMAEPNFVK--RSYNDY 79

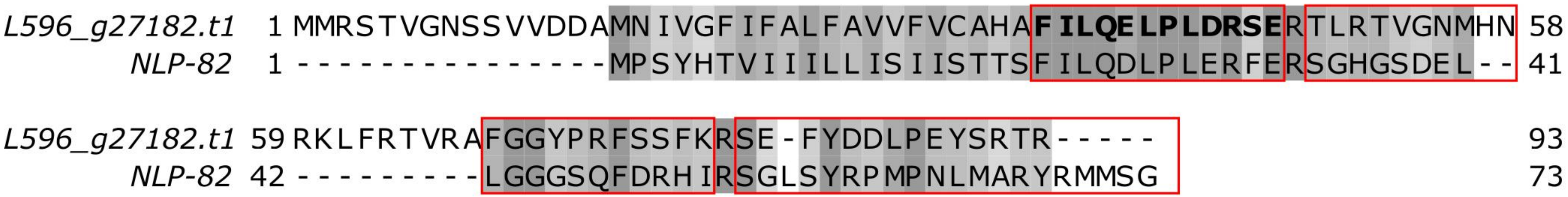
L596_g27182.t1 1 MMRSTVGNSSVVDDAMNIVGFIFALFAVVFVCAHAFILQELPLDRSERTLRTVGNMHN 58
NLP-82 1 ---------------MPSYHTVIIILLISIISTTSFILQDLPLERFERSGHGSDEL-- 41
L596_g27182.t1 59 RKLFRTVRAFGGYPRFSSFKRSE-FYDDLPEYSRTR----- 93
NLP-82 42 ---------LGGGSQFDRHIRSGLSYRPMPNLMARYRMMSG 73

```
L596_944625   1 -MKTVAVLFLLCAIVSLASSLDCRKFSFAPACRGIMLKRASSDLSDTSFDS-------   50
 Ce-NLP-83    1 MARFTPLLMILLALVPLYYSLDCRKFSFAPACRGIMLKRSGGHPMIAEQQPMIDNAKA   58

L596_944625  51 ----QFQQAISNLLQEAEQNQIECISLVWLRGKLTLSGDSVEKRSFNRFPFA        98
 Ce-NLP-83   59 REVQMMELLIRNIEDEALIANSDCVSMSWLRDRLVNAKNEMPQ----------      102
```
